# Fossil and modern penguin tarsometatarsi: cavities, vascularity, and resilience

**DOI:** 10.1111/1749-4877.12852

**Published:** 2024-06-10

**Authors:** Piotr JADWISZCZAK, Ashley KRÜGER, Thomas MÖRS

**Affiliations:** ^1^ Faculty of Biology University of Bialystok Bialystok Poland; ^2^ Department of Palaeobiology Swedish Museum of Natural History Stockholm Sweden

**Keywords:** Antarctica, Cenozoic, Sphenisciformes, tarsometatarsus, XRM‐based analyses

## Abstract

Penguin tarsometatarsi are shortened and flattened, and studies devoted to the internal characteristics of these composite bones are very limited. Therefore, we present here a comprehensive, x‐ray‐microscopy‐based analysis based on tarsometatarsi of Eocene stem Sphenisciformes from Seymour Island (Antarctic Peninsula) as well as recent *Aptenodytes forsteri*, *A. patagonicus*, and *Pygoscelis adeliae* penguins. Our study focuses on four aspects: size variability of the medullary cavities, vascularization patterns with emphasis on diaphyseal vessels, cross‐sectional anisotropy, and diaphyseal resistance to bending forces. Small‐sized Eocene penguins (*Delphinornis* and *Marambiornopsis*) show well‐developed tarsometatarsal medullary cavities, whereas the cavities of “giant” early Sphenisciformes are either smaller (*Palaeeudyptes*) or show a conspicuous intermetatarsal size gradient (*Anthropornis*). Extant penguins exhibit a decrease in cavity dimensions as their body size increases. Distributional tendencies of primary diaphyseal nutrient foramina are quite similar in the smaller *Delphinornis*, *Marambiornopsis*, and extant *Pygoscelis* on one side and in *Palaeeudyptes* and extant *Aptenodytes* on the other. *Anthropornis* shows a unique, plesiomorphic pattern with a prevalence of plantar blood supply to the metatarsals. The diaphyseal nutrient canals diverge in orientation, some obliquely away from the proximal part, others with disparate trajectories. Cross‐sectional anisotropy along the tarsometatarsal shaft generally appears to be rather low. Clustering of coherency curves along certain tarsometatarsal segments may reflect a selection process that exerts a significant influence within biomechanically crucial sections. Diaphyseal resistance to mediolateral bending forces is explicitly more efficient in extant penguins than in Eocene Sphenisciformes. This can be interpreted as an adaptation to the waddling gait of extant penguins.

## Graphical abstract

In tarsometatarsi of Eocene and extant penguins, the distribution of primary diaphyseal nutrient foramina as well as dimensions of medullary cavities appears to align more with overall body‐size categories than geological age, albeit the extinct genus *Anthropornis* is unique in both respects. Cross‐sectional anisotropy is overall low, with local clustering of coherency curves. Extant penguins exhibit more efficient diaphyseal resistance to mediolateral bending compared to their Eocene counterparts.

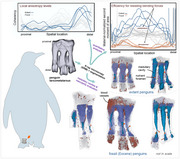

## INTRODUCTION

Among all bird orders, penguins (Aves: Sphenisciformes) have experienced the most extraordinary evolutionary shift, in terms of anatomy, physiology, and behavior, transitioning from aerial to aquatic locomotion powered by their wings (Davis & Renner 2003). They first appeared in the fossil record in New Zealand and its vicinity, dating back to the early Paleogene (specifically, the Paleocene epoch), and soon became quite diverse (e.g. Slack *et al.* 2006; Mayr *et al.* 2017; Blockland *et al.* 2019). In the case of Antarctica, the vast majority of fossil remains of early Sphenisciformes, attributable to at least 11 species (some represented by real giants), come from the Eocene strata exposed on Seymour Island, Antarctic Peninsula (Myrcha *et al.* 2002; Acosta Hospitaleche *et al.* 2019; Jadwiszczak *et al.* 2021). The stem‐group penguins persisted well into the Neogene (e.g. see phylogram in Cole *et al.* 2022: fig. 1b), and the paleontology‐informed genomic insights suggest that overall Sphenisciformes exhibit one of the lowest evolutionary rates in birds (Cole *et al.* 2022).

The hind limbs of penguins are prominently situated to the rear, an anatomical arrangement, which is well‐suited for efficient swimming and diving, for it complements their streamlined body shape. Pelvic limbs are still functionally active in the water, mainly serving (together with the tail) as a rudder (Kooyman *et al.* 1971; Harada & Tanaka 2022). The extremely posterior positioning of the legs leads to a significant osteological consequence, which is the shortening and flattening of tarsometatarsi (e.g. Myrcha *et al.* 2002). These are compound avian bones, formed as a result of fusion involving the tarsal and metatarsal components (Baumel & Witmer 1993). Their shape contributes to clumsiness while walking or running, and the associated energy cost is high due to short legs and the resultant necessity for rapid force generation (Griffin & Kram 2000). Nevertheless, it is partly compensated by the waddling gait that allows for the conservation of mechanical energy (Griffin & Kram 2000) as well as ensures stability in the frontal plane dynamics (Kurz *et al.* 2008). Additionally, balance on land may be enhanced by the extralabyrinthine sense organ of equilibrium (Jadwiszczak *et al.* 2023).

The existing studies on the internal structure of penguin tarsometatarsi, including fossil material, are very limited and have mostly utilized destructive methodology (Cerda *et al.* 2015; Ksepka *et al.* 2015; but see Jadwiszczak *et al.* 2021). The present approach to Eocene and extant penguin tarsometatarsi is aimed at a comprehensive nondestructive, x‐ray‐microscopy‐based evaluation of four crucial aspects of these extraordinary foot bones: (i) the medullary‐cavity size variability, (ii) patterns of vascularity (focusing on diaphyseal vessels), (iii) cross‐sectional anisotropy, and (iv) diaphyseal resistance to bending forces.

## MATERIALS AND METHODS

### Study subjects

**Figure 1:**
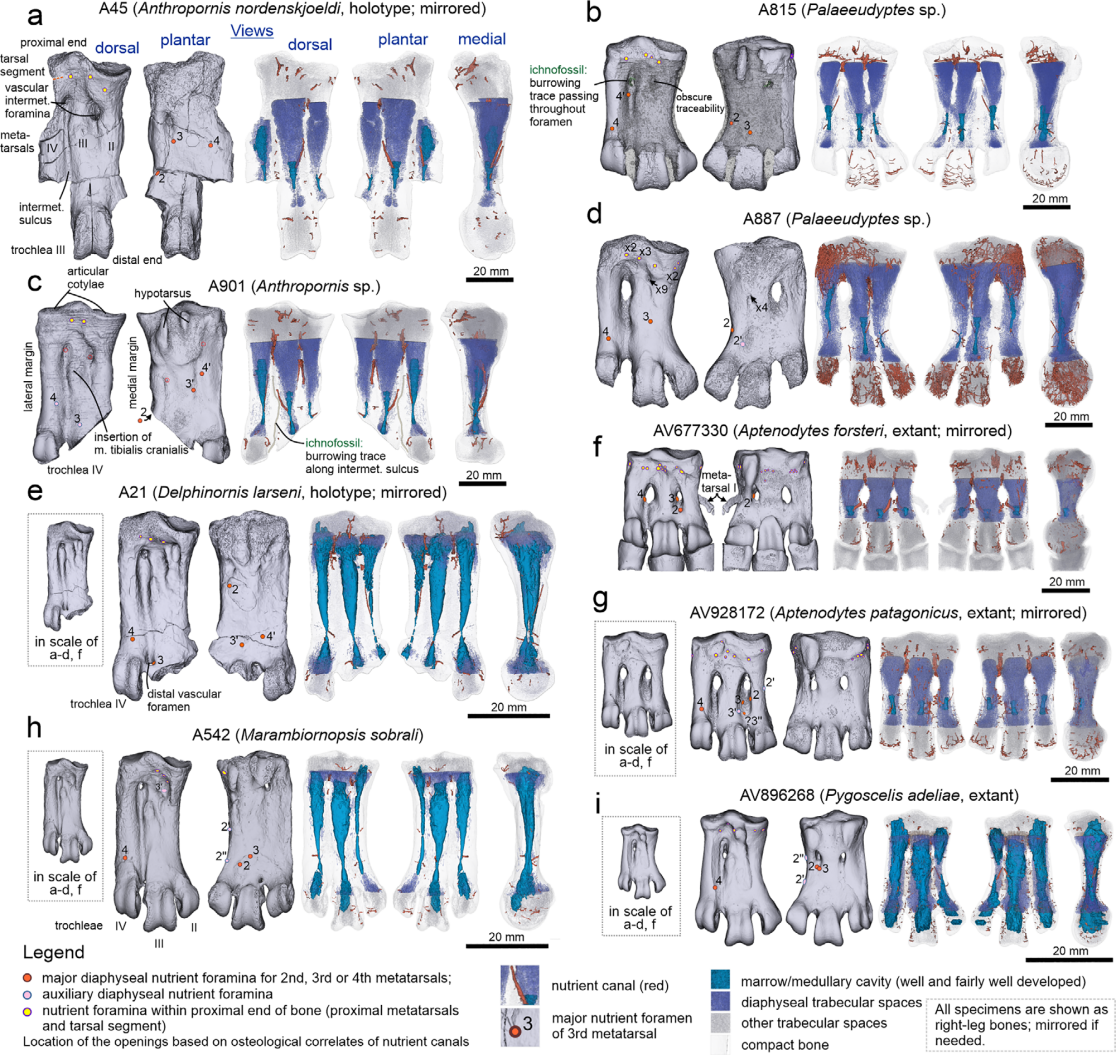
Selected tarsometatarsi of extinct (a–e,h) and extant (f,g,i) penguins representing all studied taxa, showing medullary cavities, distribution of nutrient foramina, and osteological correlates of vascularity. All specimens are presented in Fig. S1, Supporting Information.

All penguin tarsometatarsi included in the current study are housed in the paleozoological (NRM‐PZ, fossils) and zoological (NRM‐VE, extant specimens) collections of the Swedish Museum of Natural History (Naturhistoriska riksmuseet, Stockholm, Sweden). Those representing extinct Sphenisciformes are late Eocene (Priabonian) in age and were sourced from shallow marine, coastal deposits of the Submeseta Formation (TELM 7), exposed on Seymour Island (Antarctic Peninsula). The tarsometatarsi of the studied Eocene Antarctic penguins are represented by 21 specimens belonging to four different genera and at least five species (counts of specimens in parentheses): *Anthropornis nordenskjoeldi* Wiman, 1905 (holotype only), *A. grandis* (Wiman, 1905) (holotype only), and *Anthropornis* sp. (2); *Palaeeudyptes gunnari* (Wiman, 1905) (holotype only) and *Palaeeudyptes* sp. (10); *Delphinornis larseni* Wiman, 1905 (5, including the holotype); and *Marambiornopsis sobrali* Jadwiszczak *et al.*, 2021 (holotype only). Representatives of *Anthropornis* and *Palaeeudyptes* are commonly referred to as “giant” penguins (e.g. Myrcha *et al.* 2002; Cole *et al.* 2022), because they are estimated to exceed their largest contemporary counterparts in terms of body dimensions (Jadwiszczak 2001). The historical perspective of research on early Antarctic penguins is outlined in the works by Hospitaleche *et al.* (2017, 2019). The analyzed present‐day Sphenisciformes are attributable to Emperor, King, and Adelie penguins, that is, *Aptenodytes forsteri* G.R. Gray, 1844 (2, an adult and juvenile), *Aptenodytes patagonicus* J.F. Miller, 1778 (1), and *Pygoscelis adeliae* (Hombron & Jacquinot, 1841) (1). These specimens were collected in the Antarctic. Catalog numbers and visualizations for selected bones are available in Fig. 1; all of them as well as basic measurements in Fig. S1 and Table S1, Supporting Information, respectively.

### XRM data acquisition and processing

Tarsometatarsi were scanned at the Stockholm University Brain Imaging Centre, Stockholm, Sweden, using the ZEISS Xradia Versa 520 x‐ray microscope. Specimens were placed in either modified plastic containers or 50‐mm falcon tubes to be mounted to the Xradia's stage platform. Due to the considerable length of some specimens (in relation to the Xradia Versa 520's field of view), multiple scans were taken and stitched using multiple reconstructed volumes. Usually, two to three scans per specimen were enough to complete the vertical stitching. Most fossil specimens were scanned at 120 kV and 7–10 W with 1601 projections. Specimens of extant species were scanned at a lower voltage, 80 kV and 7 W with 1601 projections. Zeiss Scout and Scan software was used for scanning, reconstruction, and stitching. After these steps, stacks of TIFF‐formatted images were produced in Object Research Systems (ORS) (Montreal, Quebec Canada) Dragonfly Pro software. The resulting stacks were imported into 3D Slicer (Kikinis *et al.* 2014) for segmentation and visualization.

### Data analysis

To assess the local anisotropy within tarsometatarsal cross‐sections, we measured the orientation coherence of image features. Coherence, ranging from 0 (isotropy) to 1, was quantified for each pixel using structure tensor matrices (see Outline at http://bigwww.epfl.ch/demo/orientationj/) and extracted via the Dominant Direction function in the OrientationJ plugin (Püspöki *et al.* 2016) within the Fiji distribution of the ImageJ (Schindelin *et al.* 2012). Edges within images were enhanced using the Edges function in the FeatureJ plugin before analysis (https://imagescience.org/meijering/software/featurej/).

To investigate the resistance of the tarsometatarsal cross‐sectional shapes to bending (and torsional) forces, we focused on the second moment of the area around the principal axes of the area of bone tissue (Main 2021; Huie *et al.* 2022). The moment around the major axis quantifies the section's lowest resistance, whereas that around the minor axis represents the highest resistance. In the case of penguin tarsometatarsi, the former roughly corresponds to resistance against dorsoplantar flexing, and the latter corresponds to resistance against mediolateral (side‐to‐side) flexing (see also Huie *et al.* 2022: fig. 2). The above‐mentioned axes are mutually perpendicular and pass through the centroid of the cross‐sectional shape area. The unit of dimension of a raw value of measurement is mm^4^. To facilitate meaningful comparisons, we employed length and material normalizations. Length normalization involved dividing the fourth root of the moment by the bone length, while material normalization compared the second moment of the area with that of a solid circle with an equal area. The resulting values were unitless. For detailed information on measuring the second moment of area, refer to Huie *et al.* (2022). Calculations were performed using the SegmentGeometry plugin (Huie *et al.* 2022) in 3D Slicer.

Raw data on both local anisotropy and the second moment of the area were imported into the R environment (R Core Team 2023), and spatial changes in the parameter values were visualized using base R plotting functions. They were then graphically enhanced with the CorelDraw Graphics Suite (Corel Corporation).

## RESULTS

### Characteristics of medullary cavities

The medullary cavities in tarsometatarsi attributed to *Anthropornis* exhibit a distinctive pattern (Fig. 1a,c; Fig. S1a, Supporting Information): progressively increasing in size from metatarsal II to III and peaking at metatarsal IV. The size increase is only obscure in the case of metatarsal IV in A22 as it is damaged. This gradation appears consistent in A45, A584, and A901, with A22 showing a disproportionate size difference between metatarsals II and III. Additionally, the third cavity of A22 is notably larger than its counterparts. While metatarsal II cavities have a tube‐like shape, those in other metatarsals resemble an upturned bottle or a distally tapering funnel. In metatarsal III of A901, there is a pronounced constriction slightly beyond midway along its length.

In *Palaeeudyptes*, medullary cavities exhibit significant variation in size and shape (Fig. 1b,d; Fig. S1b–d, Supporting Information). A891 represents one end of the spectrum with three extremely large cavities, while A900, with the smallest cavities, arguably represents the other end. A heterogeneous set, including A541, A586, A594, A815, A845, A846, and A887, falls between these extremes. This collection encompasses specimens with small or moderately developed cavities. A7 and A821 present challenges. In A7, metatarsals II and III have small cavities, whereas the cavity is split into two gap‐separated parts in metatarsal IV. The small chamber is situated distally, whereas the proximal one is large in volume. In A821, the cavity of metatarsal IV is singular and very well‐developed. In most tarsometatarsi, metatarsal II has the least developed cavity, except for A845 and arguably A891. Unlike in *Anthropornis*, the cavity of metatarsal IV is often not the largest one; an extreme case is exhibited for specimen A845, in which it is the least developed. The shape of the medullary cavities is also not uniform. They are seldom funnel‐like; this is the case mainly where cavities are large, such as in metatarsal IV of A891, to a degree A7, and all metatarsals of A891. More often, they are irregularly tubular (e.g. A815, A846; branching in A900), or due to narrowing occurring roughly at a midpoint, they have the appearance of an elongated hourglass, as in metatarsals II and III of A845 and A894, and metatarsal III of A821 and A887.

Concerning the tarsometatarsi of *Delphinornis*, their medullary cavities are mostly well‐developed (Fig. 1e; Fig. S1e,f, Supporting Information). There are two potential exceptions represented by vacuities in metatarsal II in A21 (holotype of *D. larseni*) and arguably metatarsal IV in A820. However, even these two are indisputably quite distinct. In all five bones attributed to the above species, the cavity within metatarsal III appears to be the largest in volume. All metatarsals (except the two most damaged) have bipartite medullary cavities, with their parts connected by a narrow passage. These segments differ in size, with the proximal one always being much larger. The smaller chamber either reaches the base of the trochlea or extends even further distally, the latter being most conspicuous in metatarsal IV of A869. The tarsometatarsus of the holotype of *M. sobrali* (Fig. 1h; Fig. S1f, Supporting Information) has very well‐developed bipartite cavities in metatarsi III and IV (as is the case for *Delphinornis*), with their distal ends located within trochleae. The cavity for metatarsal II is mostly tube‐like along its length, its widened proximal‐most end reaching the tarsal region, whereas the opposite end terminates not far from the base of the trochlea.

In the specimen attributed to *P. adeliae* (Fig. 1i; Fig. S1g, Supporting Information), the medullary cavities of metatarsals III and IV are very large; the latter is longer and enters the tarsal region. The cavity of metatarsal II, albeit undoubtedly well‐developed, consists of two main chambers separated by a substantial gap centered at the diaphyseal midpoint. The cavities of all metatarsals extend to the respective trochleae, where those within trochleae III and IV distinctly expand in volume; in trochlea II, the structure is shorter and accompanied distally by a separate vacuity. The unsegmented cavities in metatarsals III and IV exhibit an overall shape that is influenced by a narrowing, situated approximately at their distal third. This feature is notably less pronounced than in fossil specimens.

The medullary cavities within the tarsometatarsi of *A. patagonicus* (Fig. 1g; Fig. S1g, Supporting Information) are short but not narrow, and they are positioned distally to the midpoints of their respective metatarsal shafts. The cavity in the metatarsal IV is notably more developed than in the others. They have a primarily tubular appearance, with those in metatarsals III and IV being slightly wider at their ends compared to their midpoints. The medullary cavities in the specimen attributed to an adult *A. forsteri* (Fig. 1f; Fig. S1g, Supporting Information) are even shorter than those in *A. patagonicus*, but they are positioned similarly along their respective longitudinal axes. The cavity in metatarsal IV is undoubtedly the most developed. All of these cavities have a stubby appearance, being wider proximally, and exhibit a narrowing distal to their midpoints. Considering a juvenile *A. forsteri*, its medullary cavities are notably larger in terms of both their length and width. Proximally, they extend to the midpoint of their respective metatarsal shafts, whereas distally, they enter the trochleae. Additionally, all of these cavities exhibit narrowing around their distal third.

### Distribution of diaphyseal nutrient foramina

The preserved primary diaphyseal nutrient foramina in tarsometatarsi assigned to *Anthropornis* follows a consistent pattern (Fig. 1a,c; Fig. S1a, Supporting Information). The corresponding opening within metatarsal II is positioned along the medial margin, whereas those of metatarsals III and IV are situated on the plantar surface. Among the four specimens, two (A22 and A901) exhibit accessory foramina. The former has an accessory foramen located within the medial intermetatarsal foramen; in the latter, two accessory foramina are traceable on the dorsal side of metatarsals III and IV.

When examining *Palaeeudyptes* specimens (Fig. 1b,d; Fig. S1b–e, Supporting Information), the arrangement of the described structures shows intricacy. All but A900 lack a plantar primary nutrient foramen on metatarsal II; however, the specimen possessing such a foramen, also features an additional dorsal opening. In total, six bones (A7, A541, A594, A821, A891, and A900) have their primary nutrient openings situated dorsally, whereas five specimens (A541, for which it is a second opening, A815, A845, A846, and A887) have them along the medial margin. A586 lacks the relevant portion due to damage. Six metatarsals III (A7, A541, A586, A821, A846, and A887) display these structures on the dorsal side; one of them (A846) has two such openings, and (additionally) a third one located plantarly. Two more specimens (A594 and A845) exhibit the openings in a dorsal position when considering the tarsometatarsus as a whole, but they are eccentric and noticeably shifted toward the lateral intermetatarsal sulcus. Regarding metatarsal IV, only A821 undoubtedly has the foramen on the plantar side, and it also features another one, supposedly (as it is damaged) located laterally. Seven specimens potentially display these structures in a dorsal position (A7, A541, A586, A594, A815, A887, and A891). One of them (A815) has two primary openings, another specimen (A586) has an extra accessory foramen, and the third (A586) also possesses an opening located dorsolaterally, plus one more aperture—within the lateral intermetatarsal foramen, positioned almost plantarly. Only A845 has its foramen situated solely on the lateral side, and two other bones (A846 and A900) exhibit their openings within the lateral intermetatarsal foramen.

Concerning the tarsometatarsi of *Delphinornis* (Fig. 1e; Fig. S1e,f, Supporting Information), all five of them lack nutrient foramina on the dorsal surface of their metatarsals II. Instead, the primary foramina are found either on the plantar side (A21, A820, and A861) or along the medial margin (A587 and A869). The specimen A820, in particular, exhibits two such openings on its plantar surface. Moreover, A820 has two accessory foramina: along its medial margin and inside its medial intermetatarsal foramen, and A861 possesses a single one in the former location (possibly more in the latter), whereas two auxiliary openings of A869 are positioned within the medial intermetatarsal foramen. As for metatarsal III, only A869 features its principal foramen on its dorsal side and another (auxiliary) foramen within the medial intermetatarsal foramen. Three bones exhibit these structures on their plantar surface (A21, A587, and A861); one of them (A21) is also within the distal intermetatarsal foramen. The specimen A861 features an accessory aperture within the distal intermetatarsal foramen. In the case of A820, no opening could be identified as being significant, though two foramina were present. The distribution of the primary foramina on metatarsal IV is also non‐uniform. Two specimens (A587 and A869) exclusively have them positioned dorsally, one (A21) possesses two apertures, located both dorsally and plantarly, whereas the other two have openings on either the plantar (A820) or lateral (A861) side. In the latter case, there is also some premise for a possible dorsal aperture. Only A820 possesses accessory foramina within its metatarsal IV—two located dorsally and one within the lateral intermetatarsal foramen.

The sole known specimen of *Marambiornopsis* (Fig. 1h; Fig. S1f, Supporting Information) displays the plantar positioning of the primary nutrient foramina for its metatarsals II and III, whereas the remaining one is located dorsally. There is also an accessory opening for metatarsal III, positioned within the medial intermetatarsal foramen, and some evidence of two, supposedly accessory, foramina along the medial margin of metatarsal II.

The tarsometatarsus of *P. adeliae* (Fig. 1i; Fig. S1g, Supporting Information) is characterized by the plantar location of the main diaphyseal nutrient foramina of its metatarsals II and III, positioned in close proximity to each other. These openings are situated right next to the medial intermetatarsal foramen. Moreover, metatarsal II features two secondary openings along its medial margin. The major foramen of metatarsal IV is located more distally, on the dorsal surface. The bone attributed to *A. patagonicus* (Fig. 1g; Fig. S1g, Supporting Information) exhibits all its principal diaphyseal nutrient foramina on the dorsal surface. The foramen of metatarsal II (a single opening) and those of metatarsal III (two openings, but only one that we are confident of) are positioned either at the edge or on the floor of the medial intermetatarsal sulcus. The secondary foramen for metatarsal II is located medially, whereas that for metatarsal III is situated along the edge of the medial intermetatarsal sulcus. The opening within metatarsal IV is located as in *P. adeliae*. The specimen classified as an adult *A. forsteri* (Fig. 1f; Fig. S1g, Supporting Information) resembles *A. patagonicus* in terms of the absence of key foramina on the plantar side. One of the two openings of metatarsal II and those of metatarsals III and IV are located within the intermetatarsal foramina. Another foramen of metatarsal II can be found further distally on the floor of the medial intermetatarsal sulcus. In the tarsometatarsus of a juvenile *A. forsteri*, all the foramina are positioned either close to or directly at those lengths of metatarsal edges that will frame the yet‐to‐be‐formed intermetatarsal foramina. There is a single opening within metatarsal II, metatarsal III possesses three apertures, whereas metatarsal IV has two adjacent foramina.

### Directionality of primary diaphyseal nutrient canals

In *Anthropornis*, the primary nutrient canals predominantly penetrate cortical bone obliquely, entering the medullary cavities distally to the position of their respective nutrient foramina. In metatarsals II of A22 and A901, the angle is slight, and in that of A584, the nutrient foramen is more distal to the opposite end of the canal.

The course of the primary nutrient canals is not uniform in the studied specimens assigned to *Palaeeudyptes*. The most consistent, in terms of the directionality of the above structures, is metatarsal II. In the majority of specimens, the canals traverse cortical bone at an angle, with the respective nutrient foramen being located more proximally than the entry point into the medullary cavity. However, the second primary canal of A900 and the only one of A594 are oriented in reverse, whereas A815 has it perpendicular to the longitudinal axis. Considering metatarsal III, four specimens show the canal orientation consistent with that dominant in metatarsal II (A7, A541, A821, and A887) and four in reverse (A594, A815, A891, and A900); a single bone (A846) has primary canals displaying both courses and a further two (A586 and A845) with a perpendicular alignment. Regarding metatarsal IV, three specimens have their canal orientation akin to that dominant in metatarsal II (A846, A891, and A900) and four in reverse (A594, A821, A845, and A887). Moreover, a solitary one (A815) features the canals displaying both courses; another (A586) is like the previous one, but with the third canal in a perpendicular alignment; and A7 has the structure aligned perpendicularly to the longitudinal axis. The canal course in A541 is obscure.

The integrative specimen‐wise perspective also unveils considerable variation in patterns of primary canal pathways. Assuming three simple possibilities—the nutrient foramen is more proximal than the opposite end of the canal (denoted as X), the inverted arrangement is true (Y), and there is level conformity (Z)—the following configurations are observable (metatarsals II, III, and IV, respectively): XXY (A821, A887), XYX (A891), XXZ (A7,?A541), XZY (A845), and YYY (A594). Others represent more complex arrangements involving additional principal canals:_Z[XYZ] (A586, lacking metatarsal II), ZY[XY] (A815), X[XY]X (A846), and [XY]YX (A900).

The course of the primary nutrient canals in tarsometatarsi belonging to *Delphinornis* follows a consistent pattern solely with respect to metatarsal II. In all five cases, they enter the medullary cavity distally to the position of their respective nutrient foramen/foramina. Metatarsal III exhibits marked variability in this respect—only two specimens (A861 and A869) have the proximal relative location of their nutrient foramina; the other two (A21 and A587) have both ends at similar levels; A21 also has a second canal with its orientation inverted relative to that of A861 and A869. Metatarsal IV is characterized by yet another distribution of relative positions of pairs of openings. The condition observed in metatarsal II is retained in A21 and A587. The first of them also has another canal with an inverted orientation, the only such structure in A820 and A861 is also inverted, and that in A869 shows both its ends located at similar levels.

In strict terms, none of the four tarsometatarsi that are complete enough share the same overall canal‐directionality pattern as others. Polarities of canal openings observable in A587 are subsets of those present in the respective metatarsals of A21, which has more primary canals than the former bone.

The tarsometatarsus of *Marambiornopsis* displays a set of canal orientations in which each element differs from the others. Metatarsal II exhibits the distal relative location of its principal nutrient foramen, metatarsal III— proximal, and metatarsal IV has both ends of its nutrient canal located at similar levels.

All three metatarsals of *Pygoscelis* show the proximal location of the principal nutrient foramina relative to the opposite end of the canal. In *A. patagonicus*, the polarity for the canal of metatarsal II and the only confident primary canal of metatarsal III is as above. The major nutrient foramen of metatarsal IV is located slightly more distally in relation to the opposite end of the canal. The tarsometatarsus of an adult *A. forsteri* shares a similar polarity of three main nutrient canals with *P. adeliae*; only the second such structure in metatarsal II of *A. forsteri* possesses both ends positioned almost at the same level. Considering the juvenile *A. forsteri*, the polarity of the canal openings in metatarsals II and IV is like that in *P. adeliae*. Two out of three major canals of metatarsal III have both ends located at similar levels; in the case of the third canal, the corresponding nutrient foramen is positioned slightly more proximally.

### Other observations

The nutrient foramina of proximal tarsometatarsi are primarily observable in dorsal view (Fig. 1; Fig. S1, Supporting Information), being distributed mainly along the original tarso‐metatarsal boundary or in its proximity, with varying numbers per specimen. The traceability of corresponding canals varies across specimens. In most cases, proximal rims of intermetatarsal foramina exhibit developed apertures, canals, and sinuses penetrating the interior of the proximal bone segments.

Of particular note is specimen A887, attributed to *Palaeeudyptes*, displaying a well‐preserved vascular system within the proximal end and trochleae, with over 300 isolated structures (Fig. 1d; Fig. S1d, Supporting Information). Most likely, it is a taphonomic phenomenon, as opposed to pathology. Notably, the proximal medial intermetatarsal foramen is naturally occluded, a condition also found in other *Palaeeudyptes* specimens (A821, A891, possibly A815). In A887, nine blood vessels enter the opening from the dorsal side (two subsequently merging), while four others approach from the plantar side; however, none of them appear on the opposite side (Fig. S2, Supporting Information).

The distribution of traceable trochlear blood‐vessel correlates, if sufficiently well preserved (e.g. A815), usually takes the form of a system of curvilinear branching canals running transversely, beneath the articular surface. In the case of trochlea III, they are often subdivided into medial and lateral components with only rarely marked connecting lengths at the level of the trochlear groove.

The XRM imaging of the holotype specimen of *Anthropornis grandis* (A22) revealed a distinct boundary between the tarsal component of the bone and the proximal‐most portions of the metatarsals. This boundary is not externally observable but is evident from the scan (Fig. S3, Supporting Information). This indicates that fusion has already occurred between these bone components. However, we noted that the remodeling process has not completely obliterated this boundary yet.

The body fossils considered in our work are in several cases accompanied by ichnofossils (Fig. 1b,c; Fig. S1, Supporting Information). In three instances (A815, A845, and A901), they represent burrowing traces created by small, shallow marine polychaetes or other minute vermiform invertebrates. The fourth case is most likely a double or triple bite mark left on the surface of specimen A820. The 3D shape of the two larger traces seems to indicate a vertebrate predator or scavenger.

### Local anisotropy

**Figure 2:**
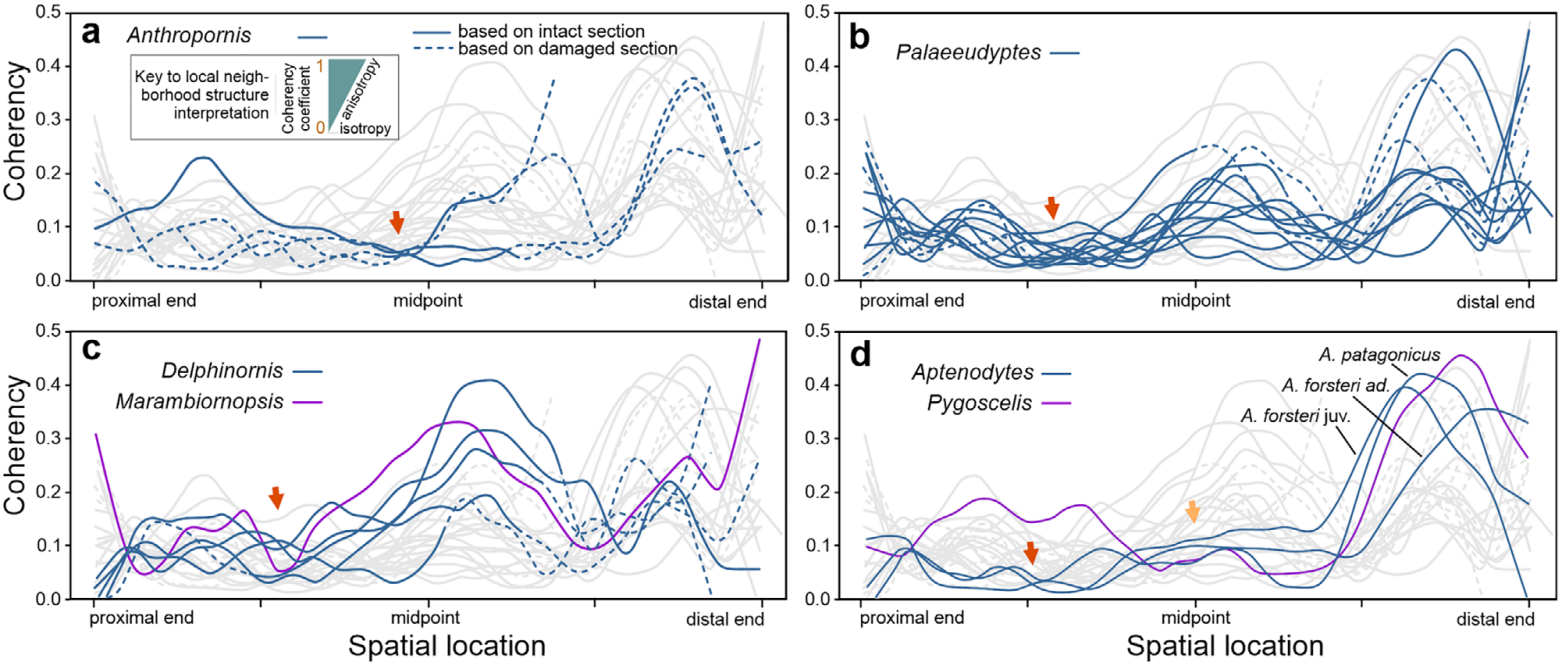
Quantitative assessment of local anisotropy in cross‐sections of tarsometatarsi attributed to (a) *Anthropornis*, (b) *Palaeeudyptes*, (c) *Delphinornis* and *Marambiornopsis*, and (d) extant genera. Coherency coefficients based on structure tensors; lines fitted using local regression (LOWESS algorithm). Arrows point at low‐anisotropy curve‐clustering sections.

The analysis of local anisotropy revealed that none of the examined tarsometatarsi, whether representing fossil or extant genera, exhibited coherency index values exceeding 0.5 (Fig. 2). In fact, these index values are typically much lower, often falling below 0.2 or even 0.1. Considering *Anthropornis*, our data on local anisotropy estimates are limited due to the condition of the available specimens. Nevertheless, there appears to be substantial variability within this set of bones. In the case of *Palaeeudyptes*, a tendency is observed with the highest anisotropy occurring particularly within their trochleae and central segments of diaphyses, while the lowest anisotropy is consistently found at the level and vicinity of the intermetatarsal foramina. Within the *Delphinornis*‐*Marambiornopsis* group, the highest anisotropy is predominantly located within the central lengths of their diaphyses, possibly also in trochleae. The lowest anisotropy values are usually observed distal to the intermetatarsal foramina. When analyzing extant genera, we found that while the highest anisotropy was present within their trochleae, the two taxa differed in terms of the location of segments characterized by the lowest anisotropy. In *Aptenodytes*, this tends to be found proximal to the intermetatarsal foramina, whereas in *Pygoscelis*, it is a feature of the central segment of the tarsometatarsus.

For all four *Anthropornis* bones, local clustering of the fitted coherency regression curves is evident. This clustering begins proximally, around one‐quarter of their length, and becomes most pronounced just before reaching mid‐length, diminishing shortly thereafter (Fig. 2a). In the case of *Palaeeudyptes*, the curves are consistently concentrated within the section spanning from one‐quarter to almost half of their length. Additionally, two localized bottleneck patterns are observed: one located near the proximal end and another at approximately three‐quarters of the length (Fig. 2b). Regarding specimens attributed to *Delphinornis* and *Marambiornopsis*, the relevant regression curves predominantly cluster along the proximal third of their course. There is also some indication of a bottleneck pattern forming at about three‐quarters of the tarsometatarsus length (Fig. 2c). In the case of the extant genera, *Aptenodytes* and *Pygoscelis*, a segment with the least variability is centered around the midpoint of bone length. However, for *Aptenodytes* alone, the fitted curves also cluster tightly along the entire proximal half of their course (Fig. 2d).

### Resistance to bending forces

**Figure 3:**
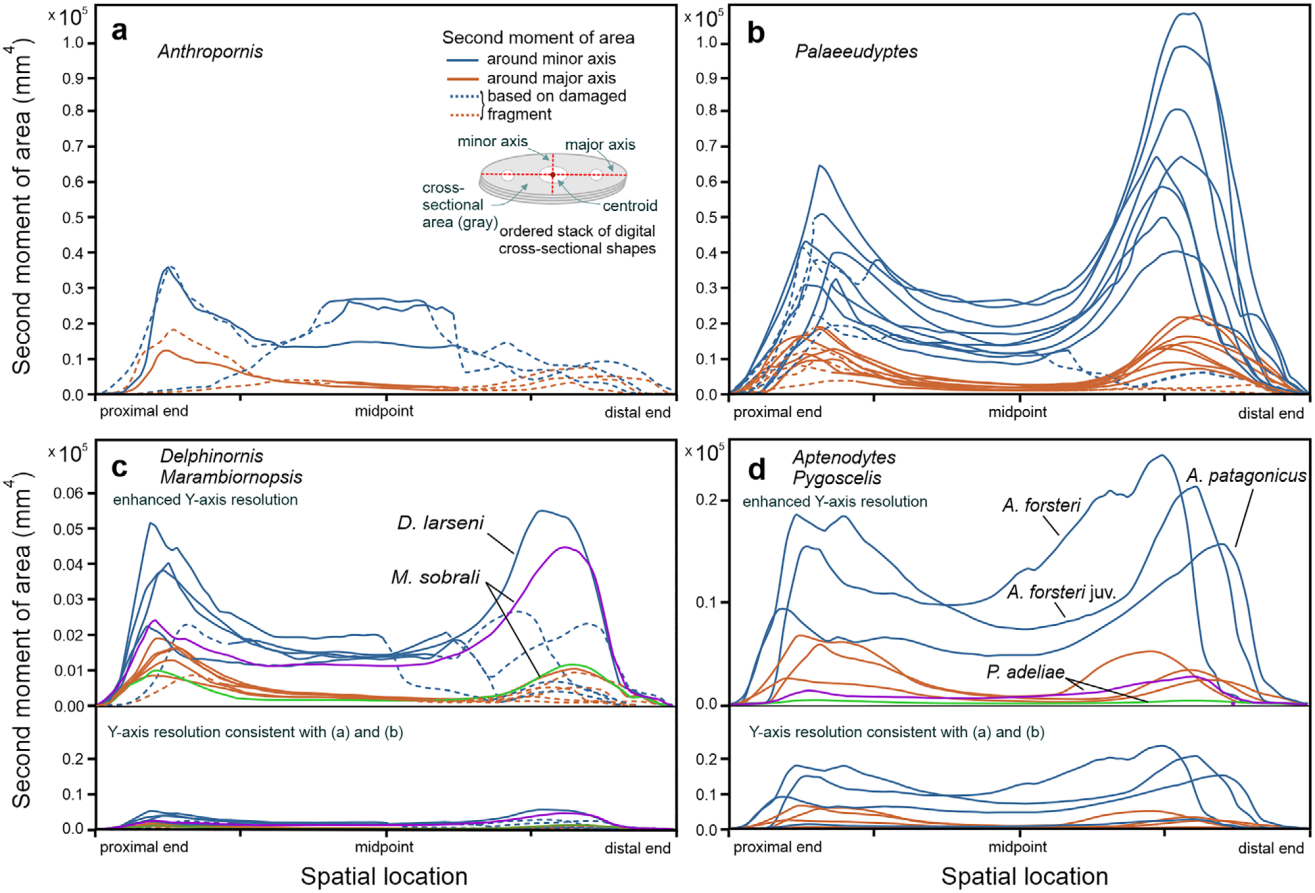
Quantitative assessment of the second moment of area *I* of cross‐sectional shapes along the length of tarsometatarsi attributed to (a) *Anthropornis*, (b) *Palaeeudyptes*, (c) *Delphinornis* and *Marambiornopsis*, and (d) extant genera. The larger the *I*, the greater the resistance of the cross‐sectional shape to bending or torsional deformation.

The analysis of resistance of diaphyseal cross‐sectional shapes to bending or flexural deformation (Fig. 3) revealed that, in absolute terms, this property was characterized by the highest (and overlapping) values in tarsometatarsi attributed to *Anthropornis* and *Palaeeudyptes*. In this respect, these two genera are followed by *Aptenodytes* (both with the juvenile *A. forsteri* included and without it)*, Delphinornis*, *Marambiornopsis*, and *Pygoscelis*. This is conspicuous for the second moment of area around the minor axis and remains true for the moment around the major axis. Moreover, the values are consistently higher for both bone extremities than for diaphyses.

**Figure 4:**
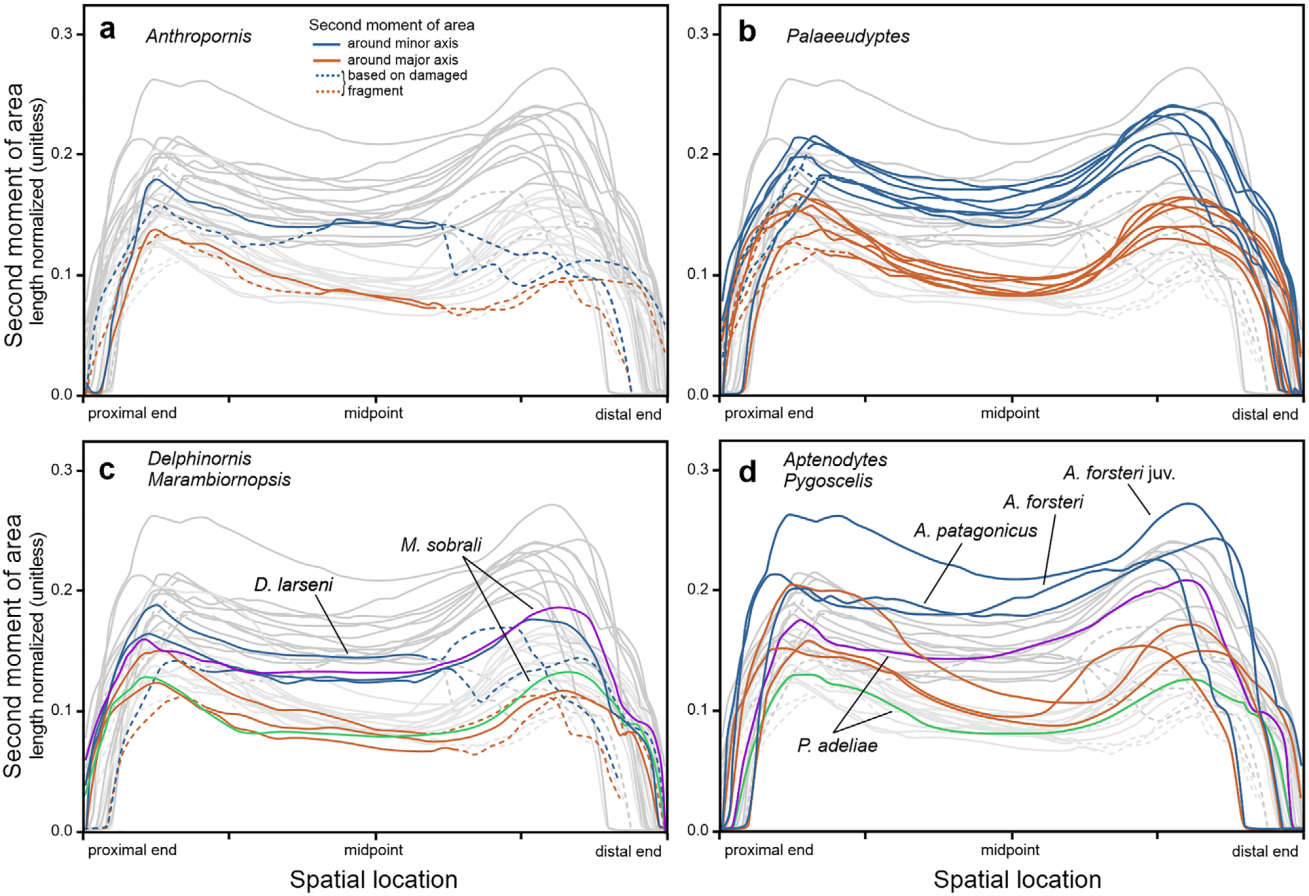
Quantitative assessment of the length‐normalized second moment of area *I* of cross‐sectional shapes along the length of tarsometatarsi attributed to (a) *Anthropornis*, (b) *Palaeeudyptes*, (c) *Delphinornis* and *Marambiornopsis*, and (d) extant genera. The larger the length‐normalized *I*, the greater the relative resistance of the cross‐sectional shape to bending.

Considering the length‐normalized diaphyseal resistance to bending forces (Fig. 4), tarsometatarsi of *Aptenodytes* penguins are, in relative terms, best able to withstand such forces acting around the minor axis. In this regard, the order for other genera is as follows: *Palaeeudyptes*, *Pygoscelis*‐*Anthropornis*, and *Delphinornis*‐*Marambiornopsis* pair. In reference to the major axis, the order is less clear, though broadly similar. Curves for adult representatives of *Aptenodytes* partially overlap with those for best‐performing *Palaeeudyptes*; the inclusion of the juvenile specimen would enhance the standing of the former genus. The remaining genera have their curves passing along lower levels, close to each other, and partially overlapping.

**Figure 5:**
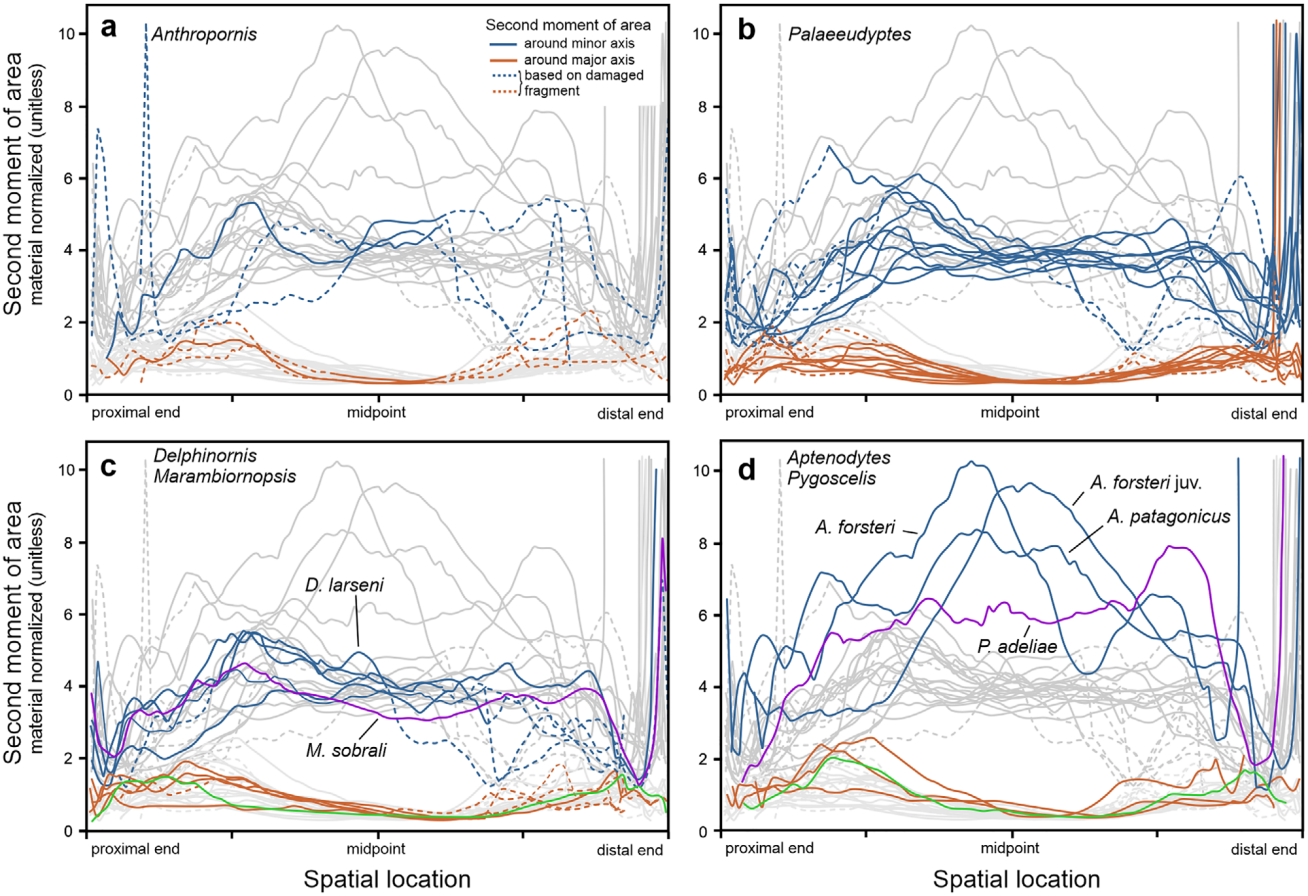
Quantitative assessment of the material‐normalized second moment of area *I* of cross‐sectional shapes along the length of tarsometatarsi attributed to (a) *Anthropornis*, (b) *Palaeeudyptes*, (c) *Delphinornis* and *Marambiornopsis*, and (d) extant genera. The larger the material‐normalized *I*, the greater the efficiency of the cross‐sectional shape for resisting bending.

Addressing the material‐normalized second moment of area, or efficiency for resistance to bending around the minor axis (Fig. 5), the most efficient along their diaphyses are extant genera, *Aptenodytes* and (to a lesser extent) *Pygoscelis*. Further down the order is *Anthropornis* followed by quite a tight cluster comprising *Palaeeudyptes*, *Delphinornis*, and *Marambiornopsis*. The last one, admittedly represented by a single specimen, seems to be overall least efficient compared to the others. Regarding the moment around the major axis, diaphyses of *Delphinornis* and *Aptenodytes*, to a lesser degree *Pygoscelis* and *Marambiornopsis*, appear to be overall more efficient than those representing *Anthropornis* and *Palaeeudyptes* in the vicinity of the midpoint of tarsometatarsal length.

In all settings presented above (Figs 3, 4, 5), the courses of (diaphyseal) curves related to the moment around the major axis are clustered much more tightly than those related to those around the minor axis.

## DISCUSSION

### Medullary cavities and some taxonomical remarks

The unequivocal indication of a distinct inner boundary demarcating the tarsal component from the proximal‐most metatarsal segments within specimen A22 (Fig. S3, Supporting Information) serves as the conclusive evidence for the relatively early ontogenetic stage. The individual was unequivocally not a chick, as metatarsals II and III are firmly fused externally and bear no indisputable signs of sutures (Fig. S1, Supporting Information), but almost certainly perished before attaining sexual maturity. The largest extant penguins attain reproductive capability at a minimum age of 3 years (Williams 1995). Moreover, A22 stands out from among the other three studied tarsometatarsi of *Anthropornis* in terms of the unusually large medullary cavity of metatarsal III and the spindle‐like shape of the medial intermetatarsal foramen (in dorsal view). These can be other markers of quite a young age, albeit we know nothing about the cavity of largely lacking metatarsal IV, and that in metatarsal II is reduced, which is typical of the genus. The above‐mentioned conditions, including incompleteness of the specimen, have the potential to raise certain concerns because the said tarsometatarsus is the holotype of *A. grandis*. As stated in the principle of typification from Article 61.1 of ICZN Code (ICZN 1999): “Each nominal taxon in the family, genus or species groups has actually or potentially a name‐bearing type.” This wording is crucial for our considerations because the type specimen (the holotype here) is the name‐bearer, not necessarily a quintessential representative (a standard for comparison) or embodiment of some species definition (Simpson 1940; Foote & Miller 2007). Simpson (1940) legitimately claimed that it is impossible for “types” to fulfill all these roles. Thus, we see no reason for challenging the current status of specimen A22.

Such reasoning can also be applied to specimen A7 (Fig. S1, Supporting Information), another holotype from the analyzed set. This name‐bearer for *P. gunnari*, one of two species from the other genus of “giant” fossil penguins, is quite abraded (especially in its proximal part) and devoid of a substantial length of fossilized tissue between metatarsals III and IV. The latter condition resulted from the influence of external factors, but it may indirectly indicate a poorly formed, hence vulnerable, floor of the intermetatarsal sulcus. This observation, together with a voluminous cavity in metatarsal IV might testify to the relatively young age of the bird. In *Palaeeudyptes*, as mentioned earlier, large medullary cavities are rather rare, and we found them in three out of 11 studied specimens. Admittedly, it cannot be ruled out that the bone damage affected the size of the cavity in one of them (A821). In contrast to this, substantial cavities are observable in some metatarsals of all four tarsometatarsi attributed to *Anthropornis*. Apart from XRM‐scanned specimens, distinct chambers are also present (via direct observation) in metatarsal IV from two damaged bones belonging to the Polish collection (Myrcha *et al.* 2002). The ground thin sections analyzed by Cerda *et al.* (2015: fig. 2) revealed the presence of “large cavities” scattered within metatarsals III and IV of *A. nordenskjoeldi*, *A. grandis*, and *P. gunnari*. Confusingly, Cerda *et al.* (2015) also noted that tarsometatarsi of both *Anthropornis* species and *P. gunnari* were composed almost entirely of compact bone, which, especially for *Anthropornis*, is not entirely in line with our (much more holistic) results. It heavily depends on the position along the shaft. The preserved metatarsals II and III of *P. klekowskii*, according to data presented by Cerda *et al.* (2015), exhibited quite well‐developed medullary cavities. Such a condition can also be found within some bones of *Palaeeudyptes* from our set. Unfortunately, the precise locations of performed cuts have not been explicitly stated by the above‐cited authors.

Considering the largest extant penguins (i.e. *Aptenodytes)* as a point of reference for the above deliberations, the bones of adult *A. forsteri* and *A. patagonicus* clearly have smaller cavities than that of a juvenile *A. forsteri*. Interestingly, the relatively small Eocene penguins (*Delphinornis* and *Marambiornopsis*) as well as extant *P. adeliae* appear to be characterized by the real substantial sizes of their medullary cavities persisting in adult individuals. In this respect, our findings related to *Delphinornis* are consistent with those of Cerda *et al.* (2015: fig. 2). The presence of distinct cavities can be directly observed (as we did) in five tarsometatarsi with damaged shafts attributed to *Delphinornis* (not only to *D. larseni*) from the Eocene penguin collection in Poland. It is also evident in plain x‐ray images of another specimen classified as *D. larseni* (Jadwiszczak & Mörs 2019: fig. 2). The medullary cavities of the specimen assigned by Jadwiszczak *et al.* (2021: fig. 3) to *Mesetaornis polaris* Myrcha *et al.* 2002, as revealed in a CT‐scan, appear to be less developed overall than those in *Delphinornis*. Our observations regarding *P. adeliae* found confirmation in a paper by Garat *et al.* (2023: fig. 2i,r).

At this point, we would like to note that modern penguins are flightless diving birds and all clues suggest that Eocene penguins were characterized by similar ecology and behavior (e.g. Williams 1995). Therefore, even when we term some of the tarsometatarsal medullary cavities as well‐developed, we are operating within the context determined by the mentioned way of life and relevant adaptations. In other words, well‐developed cavities by no means imply thin‐walled bones typical of most volant birds. Tarsometatarsi have not been covered in important papers on penguin histology by Meister (1962) and Ksepka *et al.* (2015). Nevertheless, Ksepka *et al.* (2015) claimed that the hind limb bones (only the femur and tibiotarsus were studied) had likely gained modern levels of compacta thickness earlier in penguin evolution than those from the wing skeleton. They also reported that said bones in early Sphenisciformes had smaller medullary cavities than those in their living counterparts. The latter generalization does not hold true in the case of tarsometatarsi, and observed patterns are more complicated (Fig. 1; Fig. S1, Supporting Information).

### Diaphyseal vascularity

The estimates for regional blood flow rates, governed by the energy requirements of tissues, and sizes of diaphyseal nutrient foramina appear to be correlated in the long bones of birds. However, regarding the leg, the specific objects of interest for researchers are femora (Allan *et al.* 2014; Hu *et al.* 2022). In compound bones with retained separate medullary cavities, such as penguin tarsometatarsi, blood supply (and drainage) to a large extent reflects this complexity. The resulting existence of three sets of independent foramina and canals as well as variation in their numbers complicates physiological modeling. The nutrient arteries are not the only sources of blood, albeit supposedly the most important ones. They are locally supplemented by epiphyseal vessels, and the diaphyseal cortex is also penetrated by periosteal vessels (Fig. 1b,d; Allan *et al.* 2014).

The growth of avian metatarsals is asymmetric, with greater elongation occurring at their proximal ends during embryological development. One of the consequences is a distal position of the hallux observed in adult birds, despite it initially being located at mid‐length of the (early) embryonic metatarsal II (Botelho *et al.* 2017). One can postulate that, by extension, a similar mechanism can be, at least partially—because of anatomical and biomechanical constraints—responsible for oblique courses of nutrient canals in long bones. Such orientation is fairly typical of human long bones, though frequent deviations from this pattern have been observed in tetrapods (Hughes 1952). Our investigations revealed that some of the studied diaphyseal nutrient canals were conspicuously directed obliquely away from the proximal part of the bone, but others exhibited disparate trajectories. Importantly, differences are observed within and among specimens. According to Hughes (1952), nutrient‐canal trajectories are likely a product of forces exerted by both the growing bone and growing limb vessels. In our view, it is indeed reasonable to suggest that both mechanical and vascular factors play a role in shaping the discussed patterns, albeit precise mechanisms may vary.

Considering the entire analyzed set and tarsometatarsi treated as integrated entities with two large common surfaces and two broad margins, the observed patterns are in fact tendencies or trends. In *Palaeeudyptes*, the most abundantly represented genus, the largest number of main diaphyseal nutrient foramina are observed on dorsal tarsometatarsal surfaces. The primary foramina are less frequent, but not uncommon, on their plantar surfaces as well as both margins. Such a distribution is also observable in specimens classified as *Aptenodytes*. This pattern may be indicative of the prevalence of the dorsal blood supply to metatarsals and is consistent with a known distribution of main blood vessels within penguin hind limbs, distinguished by two dorsal metatarsal arteries (Watson 1883; Zuckerkandl 1895; Midtgård 1982; Jadwiszczak 2015: fig. 8b).

Tarsometatarsi of *Anthropornis* penguins exhibit a different setting, as there exists no obvious primary nutrient foramina on their dorsal surfaces. The main nutrient vessels for metatarsals III and IV (possibly also for metatarsal II) originated from two plantar arteries, branches of the above‐mentioned dorsal vessels, which passed through intermetatarsal foramina and run in a distal direction along the plantar surface (Jadwiszczak 2015: fig. 8b). The presence of presumably three main diaphyseal nutrient foramina on the plantar surface of the tarsometatarsus of Paleocene *Waimanu manneringi* Jones, Ando et Fordyce, 2006 from New Zealand, conspicuous in available sources (e.g. Mayr *et al.* 2017: fig. 1f), seems particularly informative in the above context. *W. manneringi* is the oldest known fossil penguin with an elongated tarsometatarsus, which is a plesiomorphic trait (e.g. Mayr *et al.* 2017).

Tarsometatarsi attributable to *Delphinornis*, *Marambiornopsis*, and extant *Pygoscelis* appear to represent a different model, with metatarsal IV supplied mainly dorsally, metatarsal III mainly plantarly, and metatarsal II either plantarly or medially. Interestingly, the above clustering of taxa indicates that the phylogenetic signal is rather not prevalent here, and at the root of variation, there may lie other factors, for instance, size differences. Midtgård (1982) had studied the hind‐limb vascular system in as many as 43 species of birds, including a single penguin species (extant Peruvian penguin, *Spheniscus humboldti* Meyen, 1834), and concluded that such patterns ought to serve as merely additional characters in phylogenetic considerations.

### Local anisotropy

Bones are mechanically anisotropic (Currey 2006), meaning that their strength, stiffness, and other mechanical properties vary with direction. These characteristics are closely related to the microstructure and arrangement of both mineralized matrix and collagen fibers. Therefore, bone structural anisotropy serves as a proxy for its mechanical properties, which is particularly important when studying fossilized specimens. The clustering of coherency curves along certain tarsometatarsal segments, revealed by our study (Fig. 2), likely indicates that these specific lengths of bone are subjected to similar biomechanical constraints. Interestingly, this pattern tends to be linked to low values of coherency coefficients, that is, insubstantial anisotropy. The mentioned values are often much lower than 0.1, which allows one to conclude that there is virtually no preferred structural direction in the local neighborhood (Püspöki *et al.* 2016). On the contrary, sections with relatively higher values mostly exhibit quite considerable vertical scatter of coherency curves across specimens. A plausible explanation for these contrasting characteristics is that natural selection exerts a notably strong influence within biomechanically crucial sections, whereas constraints are less restrictive in others. A necessary complement to the above proposal is the observation that bone microarchitecture aligns spatially in the direction of the most frequently experienced stresses to withstand typical loads (e.g. Hart *et al.* 2017). In the case of diaphyses of main hind limb bones, there is undeniably effective transmission of compressive loads along the main axis and low anisotropy observed in (transverse) cross‐sections along the shaft. According to Currey (2003), substantial stresses directed radially in the endosteal–periosteal direction are infeasible. Hence, low anisotropy appears to be either a product or (more precisely) a by‐product of selection pressure as well.

In “giant” fossil penguins attributed to *Anthropornis*, the particularly selection‐driven condition appears to coincide with the pronounced convexity along the medial margin of the tarsometatarsal shaft. It is a diagnostic trait for this genus (e.g. Myrcha *et al.* 2002), interpreted as a supportive structure (Jadwiszczak & Mörs 2011), but its osteological origin remains uncertain. Penguin limb bones have been described as either pachyostotic (Meister 1962), osteosclerotic (Ksepka *et al.* 2015), or pachyosteosclerotic (Houssaye 2009; but see Houssaye *et al.* 2016). Considering the convexity, we lean toward its largely pachyostotic roots (i.e. a hyperplasy of the periosteal cortex). In *Palaeeudyptes*, another genus represented by extremely large‐sized extinct Sphenisciformes, such a supportive structure does not exist and tarsometatarsi are usually less elongated (Myrcha *et al.* 2002; Jadwiszczak & Mörs 2019). The strongest influence of natural selection is exerted within the section characterized by the weakest fusion of metatarsal diaphyses, specifically at the level of intermetatarsal foramina and immediately distal to them. The term stress‐transmission bottleneck seems appropriate here. The above observation is essentially true also for much smaller though more elongated tarsometatarsi assigned to *Delphinornis* and *Marambiornopsis*.

Within the broadly construed tarsometatarsal shafts of *Palaeeudyptes*, *Delphinornis*, and *Marambiornopsis*, segments with higher values of anisotropy tend to correspond to regions with the strongest fusion of metatarsals. This is not obvious for *Aptenodytes*, and this certainly does not apply to *Pygoscelis*. In the case of both contemporary genera, the curves of anisotropy values in the proximal third differ dramatically from one another. In our opinion, the main cause of such a condition is a clear disparity in proportions. Addressing pronounced anisotropy in tarsometatarsal trochleae, this phenomenon likely results from microstructural adaptation to the specific demands placed on these elements, such as changes in load distribution within metatarsophalangeal joints acting mainly in a single plane.

### Resistance to bending forces

The primary function of vertebrate long bones is, as expressed by Currey (2006), to apply forces to the surroundings, necessitating their ability to endure significant bending moments. However, withstanding them ought to be achievable taking account of a significant constraint—the need for material mass minimization. de Margerie *et al.* (2005) reported, based on limb‐bone analysis of 22 avian species, that tarsometatarsi were among those skeletal elements that predominantly exhibited structural properties that resist bending and axial loads. The above‐mentioned properties include, but are not limited to, relatively thicker walls and less circular cross‐sectional shape. According to Main *et al.* (2021), for resisting axial compression and tension “any bone shape will do,” and this (simplified) view results from the observation that arising stresses are influenced by the cross‐sectional area (stress being force per area). All other conditions being constant, thicker cortical bone can better distribute and withstand compressive forces. While bearing in mind that bones are seldom subjected to a single type of loading (e.g. Main *et al.* 2021), there appears to be wide consensus that prevalent forces in vertebrate limb bones primarily consist of bending and torsional loads (see Habib & Ruff 2008).

Sphenisciformes, as articulated by Davis and Renner (2003), are birds living in two worlds. In both of them, their tarsometatarsi, contributing to the structure and function of the foot, are subjected to bending and torsional forces. While on land or ice, especially during the breeding season, penguins can use their feet extensively, at times covering remarkably large distances, often across rough and steep terrain (e.g. Williams 1995). Admittedly, they spend most of their lifetime at sea, and the utilization of their feet as rudders and brakes in underwater swimming and diving has long been recognized (e.g. Evans 1900; Kooyman *et al.* 1971). Harada and Tanaka (2022) incisively remarked that the moment arm from the body center was larger (i.e. more advantageous biomechanically) for the feet (and the tail) than for wings in pitch and yaw motion (i.e. around the transverse and vertical axes respectively), though not in roll torque.

The second moment of area is a convenient and widely used tool to investigate the mechanical adaptations of biological structures (Huie *et al.* 2022). It is important to note that its utility is not limited to considerations touching flexural forces, but can be easily extended to encompass capacity to resist torsional loads. The relevant measure is called the polar second moment of area *J*, and for a given cross‐sectional shape, it is simply the sum of the second moments of area around its major and minor axes (Marelli & Simons 2014).

In the context of inner workings, the second moment of the area involves the quantification of how the material within a cross‐sectional shape is distributed to resist bending forces (Huie *et al.* 2022). Since it is calculated by integrating the square of the distance between each infinitesimal area element and a chosen axis over the entire cross‐sectional area, the observed gradient of genera (Fig. 3) is self‐evident: the more massive the bone the greater the moment. However, this holds true with absolute values but may change when introducing some common denominator (*sensu lato*). Indeed, the transition to relative values has proven significantly informative.

Length normalization, by decreasing the effect of longitudinal dimension, revealed a new ordination of penguin tarsometatarsi based on their diaphyseal resistance to bending (Fig. 4). In relative terms, both largest extant Sphenisciformes appear to be better adapted to withstand such forces than all the remaining ones, regardless of their size and geological age. The difference is quite obvious around the minor axis (running approximately dorsoplantarly), but disputable around the major axis (running more or less mediolaterally). The above comparison pertained to the joint influence of three factors: cross‐sectional shape, its size, and material investment (see Huie *et al.* 2022). When compared with data from a sample of other birds (of different sizes and locomotion modes; Doube *et al.* 2012), length‐normalized maximum diaphyseal second moment of area (*I*_max_, corresponding to *I*_minor_ in Huie *et al.* 2022) for Sphenisciformes in this study proved to be much larger. Its values are clearly below 0.1 in tarsometatarsi from the former group, even that of extinct moa, *Emeus crassus* (Owen, 1846), whereas they decidedly exceed 0.1 in all penguins.

Considering material normalization, one is focusing on the cross‐sectional shape (or “design”) itself, specifically, its efficiency in resisting flexion. In this regard, diaphyseal cross‐sections of both studied extant genera turned out to be theoretically best at enduring bending forces around the minor axis when compared to area‐compatible solid circles (Fig. 5). They seem to perform a task using the most efficient structural design, which means being optimized to be as resistant to deformation as possible while using the least amount of material.

Regardless of the employment of normalization, the estimated resistance to mediolateral diaphyseal deflection consistently exceeds that of flexural forces acting in a perpendicular direction. This is chiefly a consequence of the pronounced shortening and (particularly) dorsoplantar flattening of penguin tarsometatarsi in response to evolutionary processes shaping the bodies of these extremely specialized seabirds. Indeed, biomechanical constraints imposed on hind limbs, especially feet—body parts acting as rudders and brakes—in connection with effective wing‐propelled underwater locomotion, have been profound. We are convinced, though, that there is also some virtually overlooked terrestrial facet of this trait. The waddling gait of extant penguins generates substantial body motion in the frontal plane, and we have already mentioned that there are certain biological advantages of such dynamics (see Griffin & Kram 2000; Kurz *et al.* 2008). Nevertheless, this side‐to‐side swaying certainly results in the non‐negligible or maybe even quite significant bending forces acting mediolaterally. Sphenisciformes are essentially digitigrade when walking or running (based on our personal observations; see also Simpson 1946); hence, the tarsometatarsi (due to their location) play a crucial role in transferring forces between pedal phalanges and more proximal long bones. In such a setting, the large second moment of the area around the minor axis is advantageous.

## CONCLUSIONS

It appears justifiable to claim that small‐sized Eocene penguins (*Delphinornis* and *Marambiornopsis*) are characterized by well‐developed tarsometatarsal medullary cavities, whereas these structures in their counterparts of “giant” early Sphenisciformes are rather genus‐specific and tend to be either smaller (*Palaeeudyptes*) or exhibit a conspicuous intermetatarsal size gradient (*Anthropornis*). We also found some indication that cavity size was inversely related to body dimensions in extant penguins. This observation makes the foundation of the aforementioned specificity all the more unclear.

Distributional tendencies of primary diaphyseal nutrient foramina appear to be thought‐provoking as well. *Palaeeudyptes* and extant *Aptenodytes* are quite similar to one another in this respect, as do smaller *Delphinornis*, *Marambiornopsis*, and extant *Pygoscelis*. *Anthropornis* are of special interest here, because their unique pattern seems to be plesiomorphic, as it is also observable in Paleocene *Waimanu* and suggestive of the prevalence of plantar blood supply to metatarsals.

The cross‐sectional anisotropy along the tarsometatarsal shaft appears to be mostly rather low in all specimens, which is essentially expectable. However, observable clustering of coherency curves along certain tarsometatarsal segments is quite evident and may reflect the natural selection that exerts a notably strong influence within biomechanically crucial sections.

Considering the diaphyseal resistance of tarsometatarsi to bending forces acting mediolaterally, extant penguins are explicitly more efficient than Eocene Sphenisciformes. This can be an adaptation to the waddling gait.

## Supporting information


**Figure S1** All studied tarsometatarsi of Eocene and extant penguins.

**Figure S2** Specimen A887 (*Palaeeudyptes* sp.) showing lack of observable functional patency of the medial intermetatarsal foramen. Different blood vessels penetrate the foramen dorsally and plantarly.

**Figure S3** Evidence of the young age of the penguin represented by specimen A22 (*Anthropornis grandis*, holotype). Specimen A45 represents an adult individual assignable to *Anthropornis nordenskjoeldi*; notice the obliteration of the tarso‐metatarsal boundary.

**Table S1** Measurements of studied penguin tarsometatarsi
