# Supplementary material for: Fossil and modern penguin tarsometatarsi: cavities, vascularity, and resilience
Source: Integr Zool. 2024 Jun 10;20(3):551–67. doi: 10.1111/1749-4877.12852 (PMC12046465; doi:10.1111/1749-4877.12852)

Figure S1 All studied tarsometatarsi of Eocene and extant penguins.

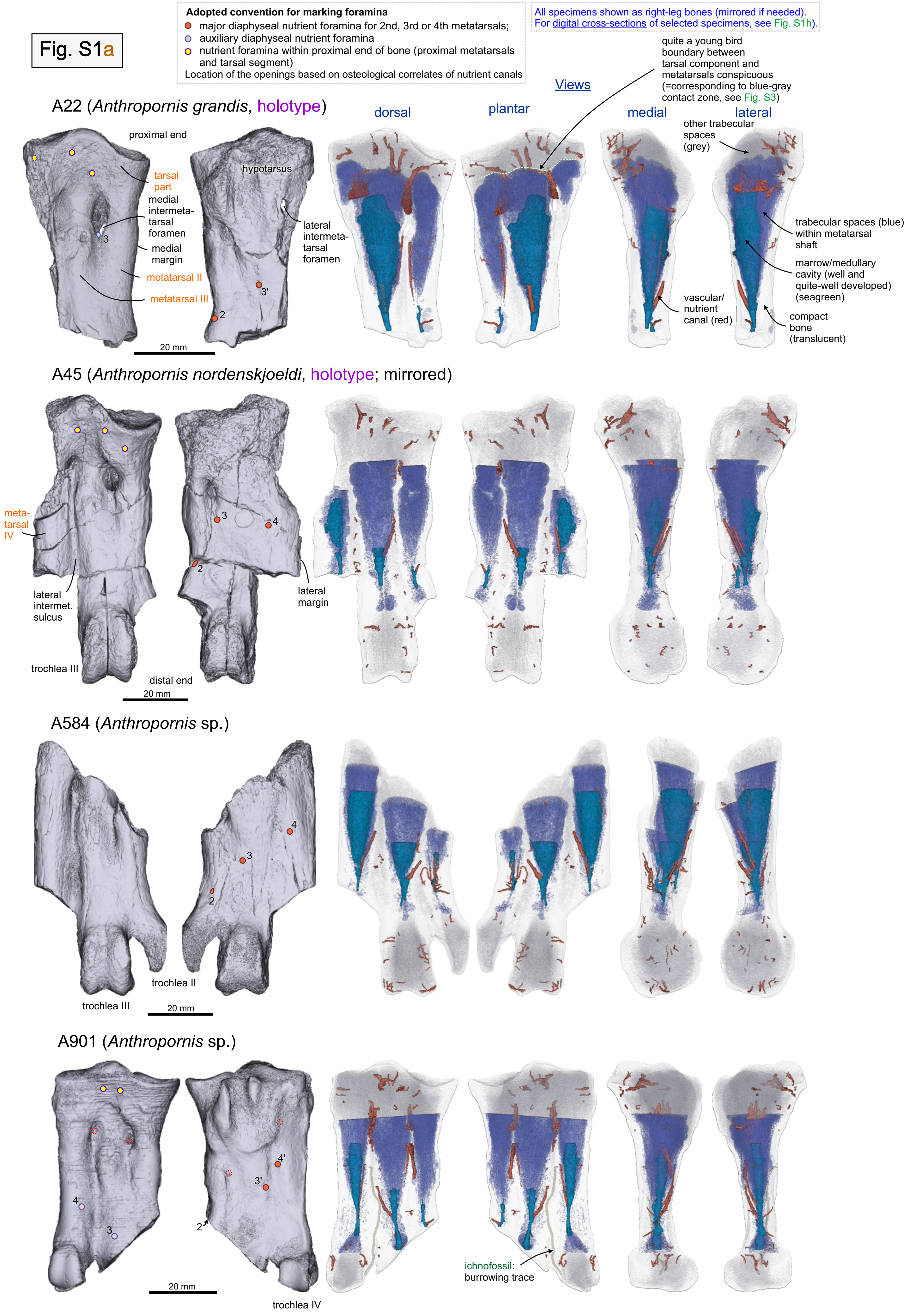

Fig. S1b

A7 (*Palaeeudyptes gunnari*, holotype)

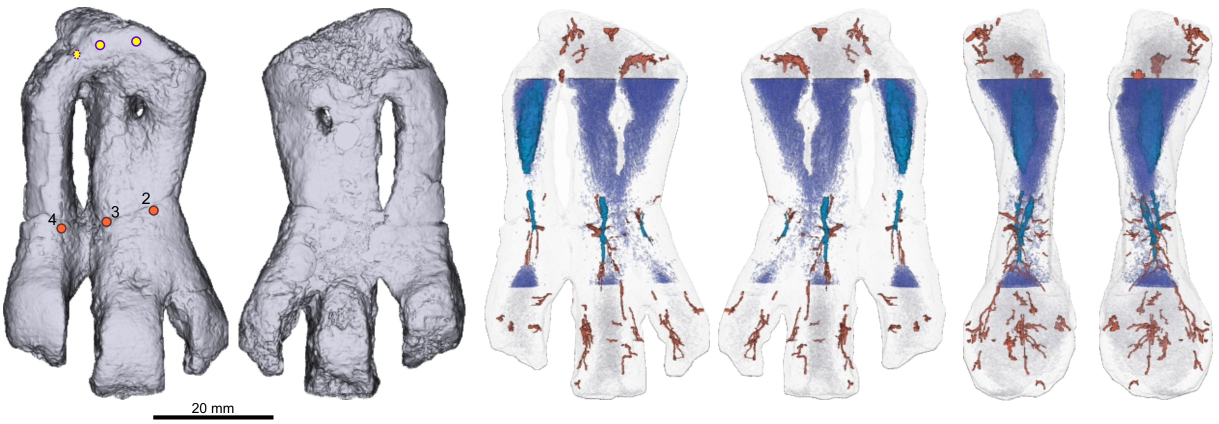

A541 (*Palaeeudyptes* sp.)

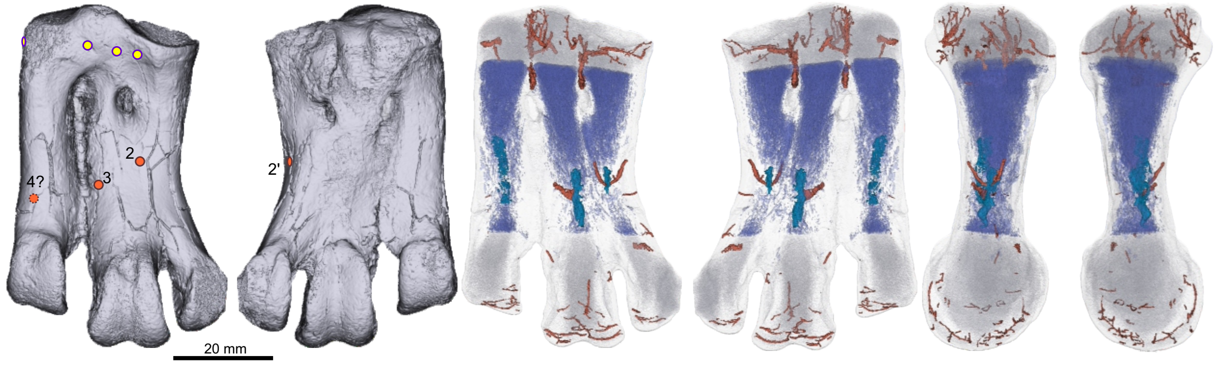

A586 (*Palaeeudyptes* sp.; mirrored)

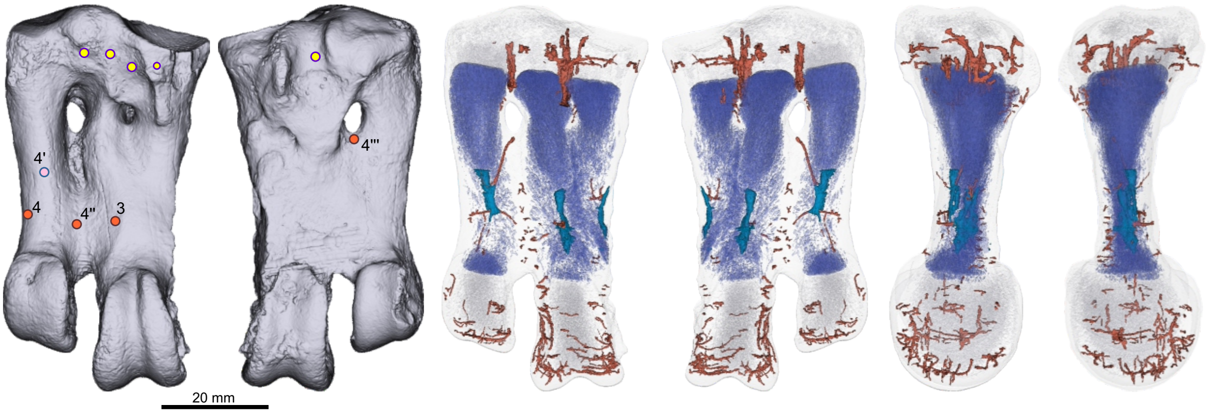

A594 (*Palaeeudyptes* sp.)

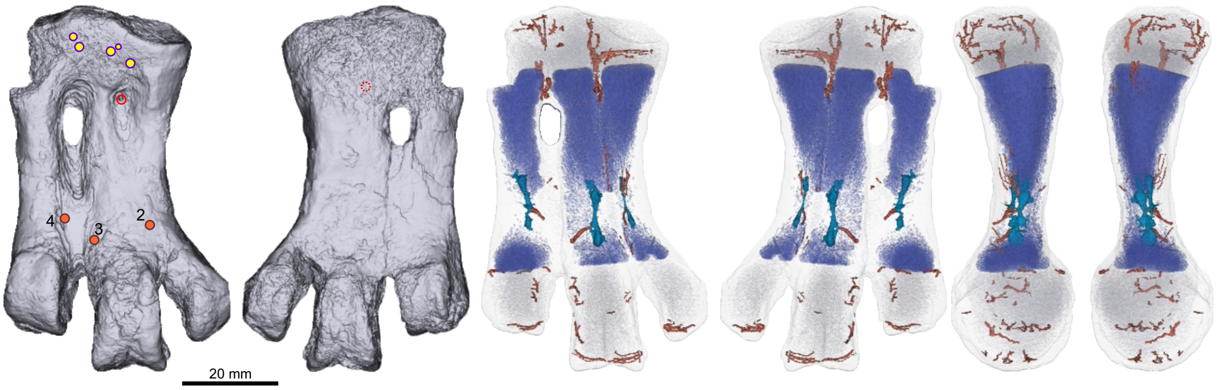

Fig. S1c

A815 (*Palaeeudyptes* sp.)

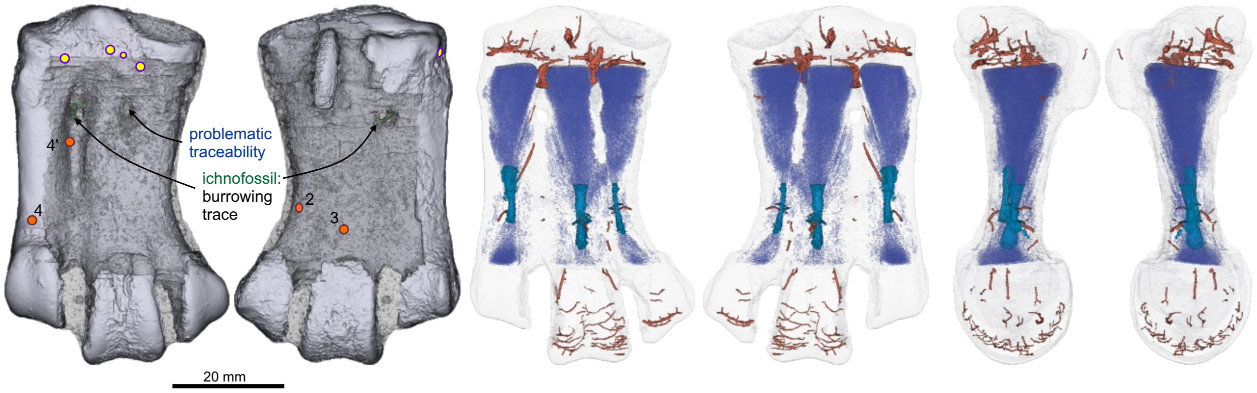

A821 (*Palaeeudyptes* sp.; mirrored)

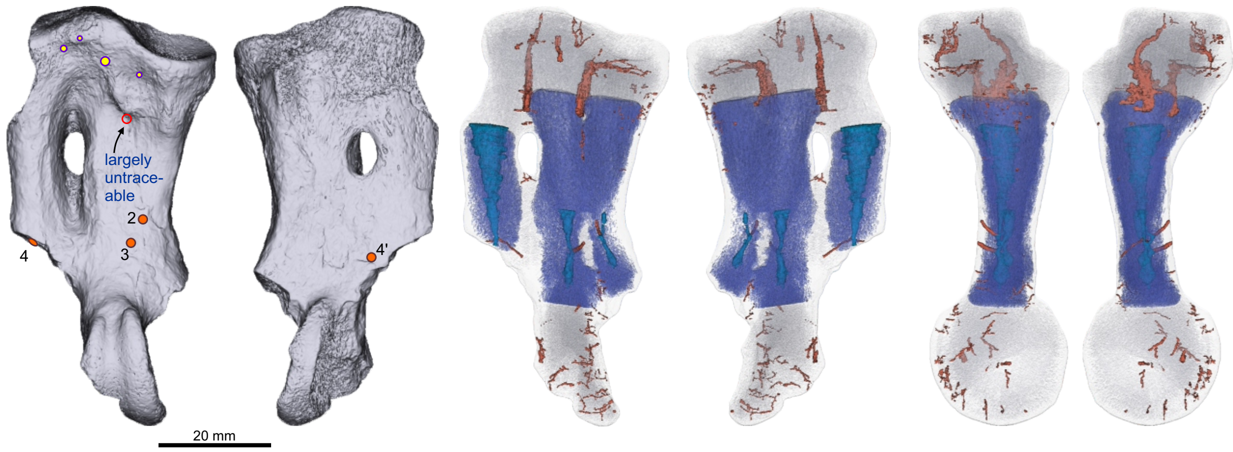

A845 (*Palaeeudyptes* sp.)

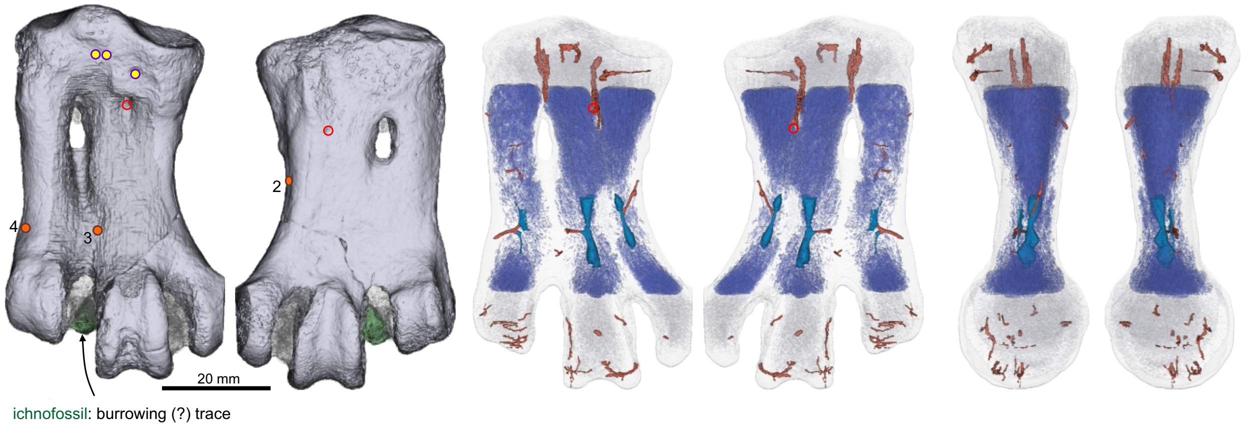

A846 (*Palaeeudyptes* sp.)

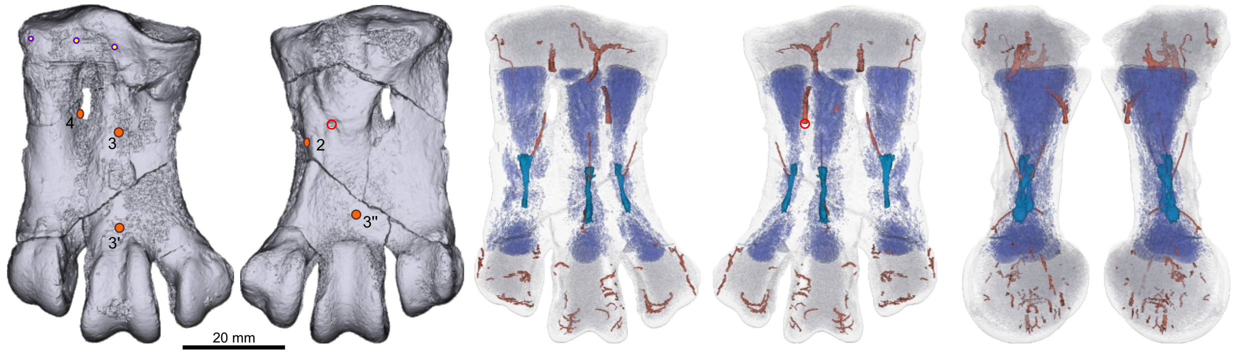

Fig. S1d

A887 (*Palaeeudyptes* sp.)

extremely well-preserved
correlates of vascular system

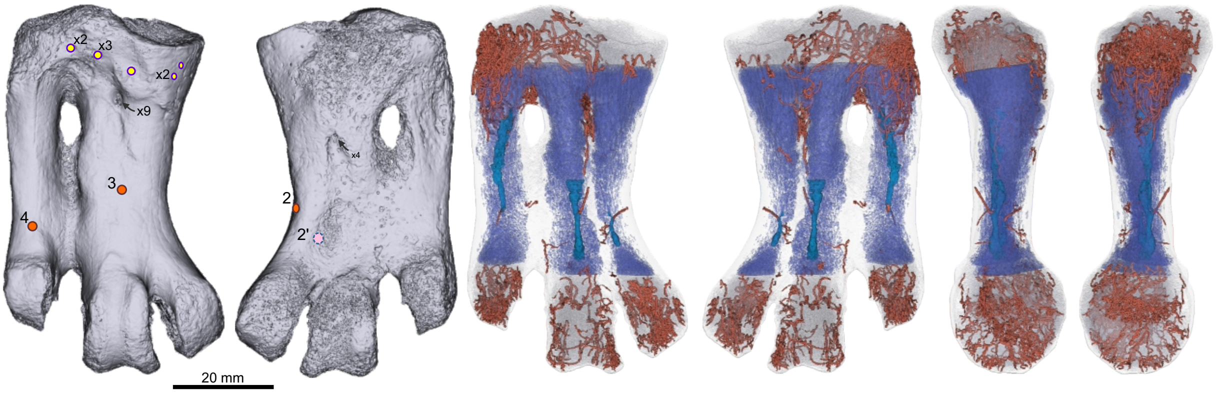

A891 (*Palaeeudyptes* sp.; mirrored)

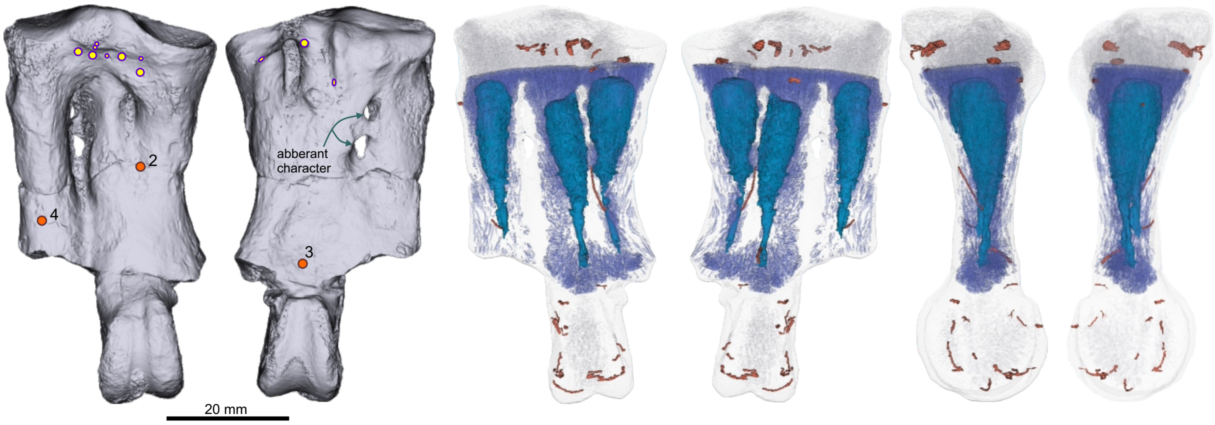

A900 (*Palaeeudyptes* sp.; mirrored)

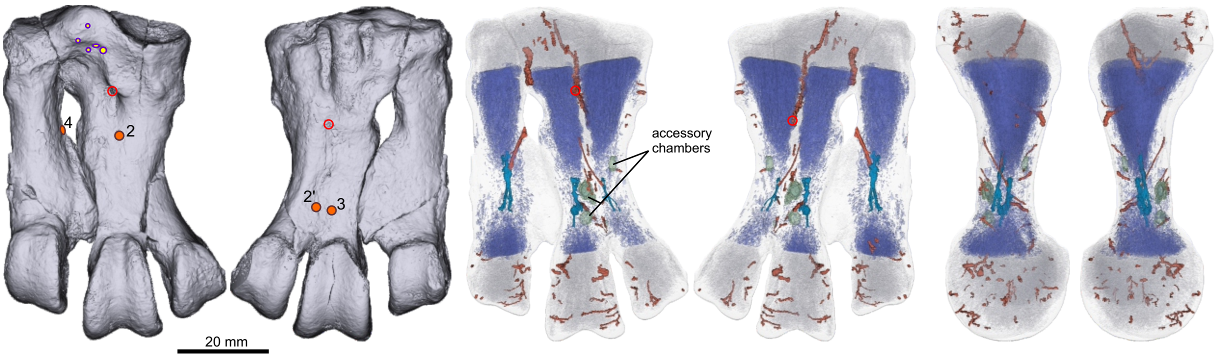

Fig. S1e

A21 (*Delphinornis larseni*, holotype; mirrored)

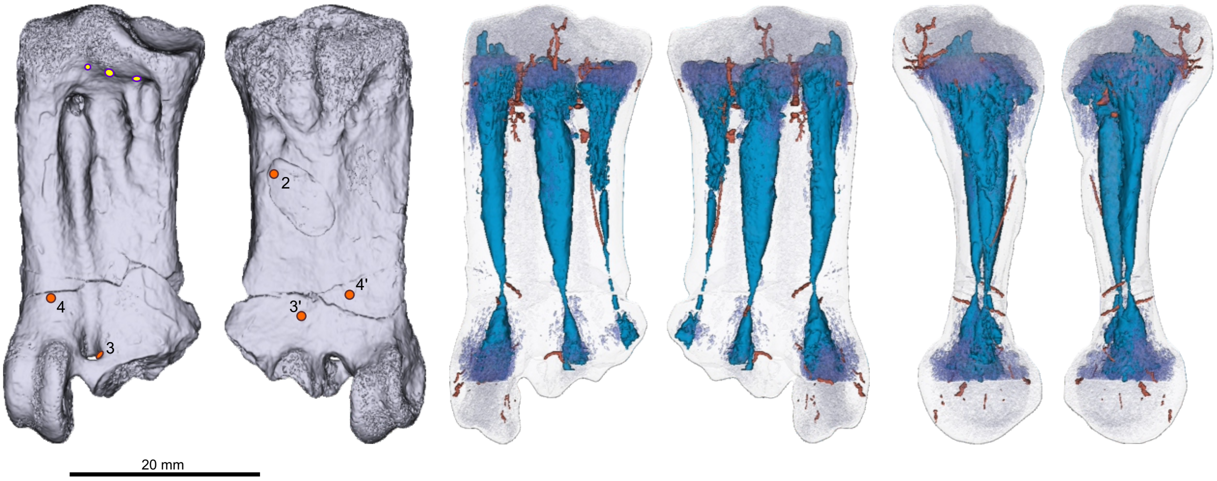

A587 (*Delphinornis larseni*)

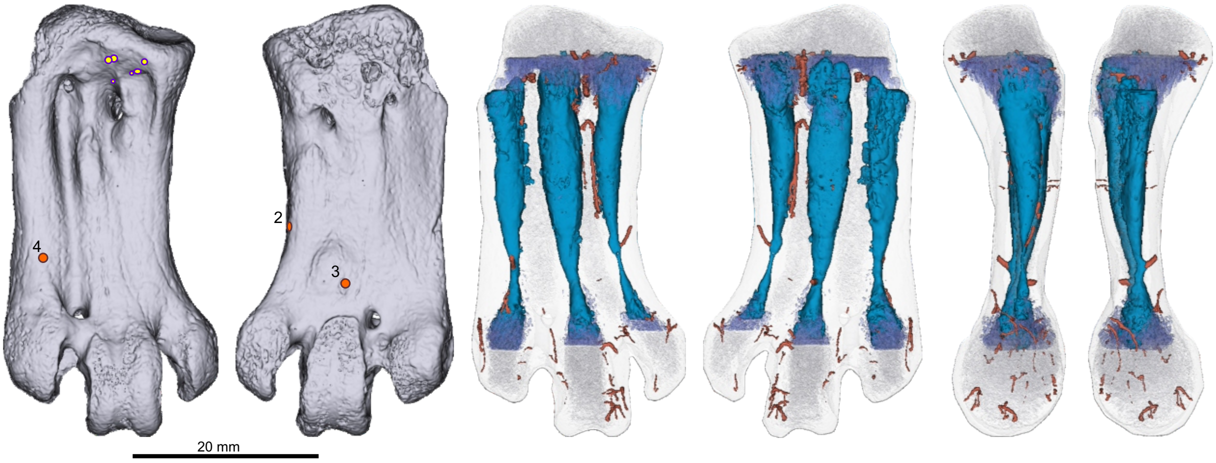

A820 (*Delphinornis larseni*; mirrored)

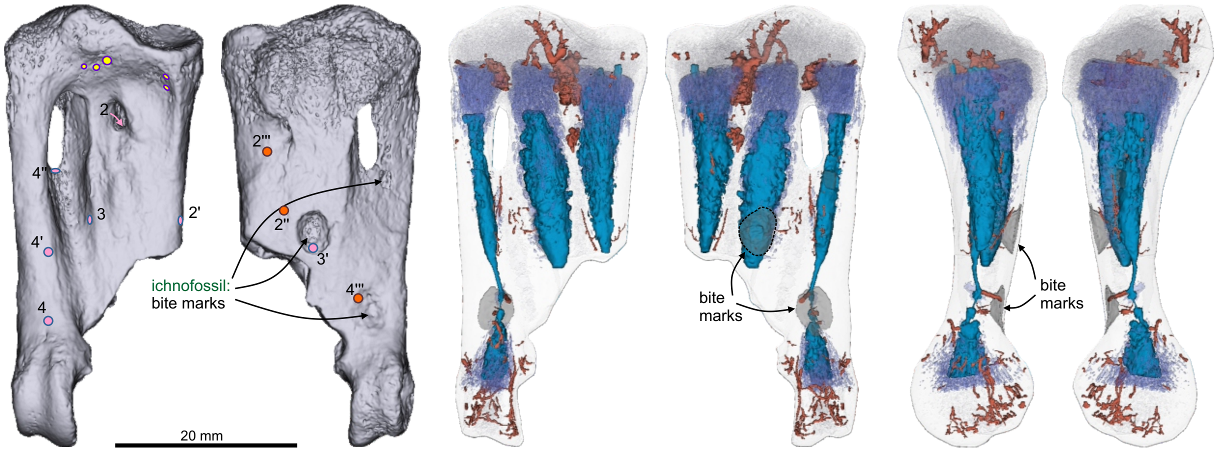

Fig. S1f

A861 (*Delphinornis larseni*; mirrored)

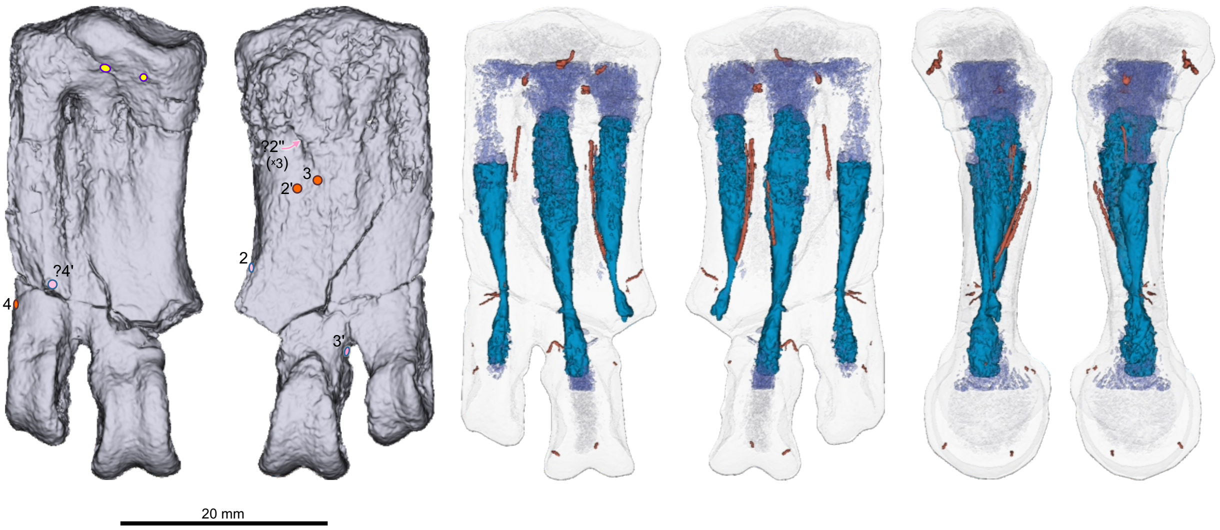

A869 (*Delphinornis larseni*)

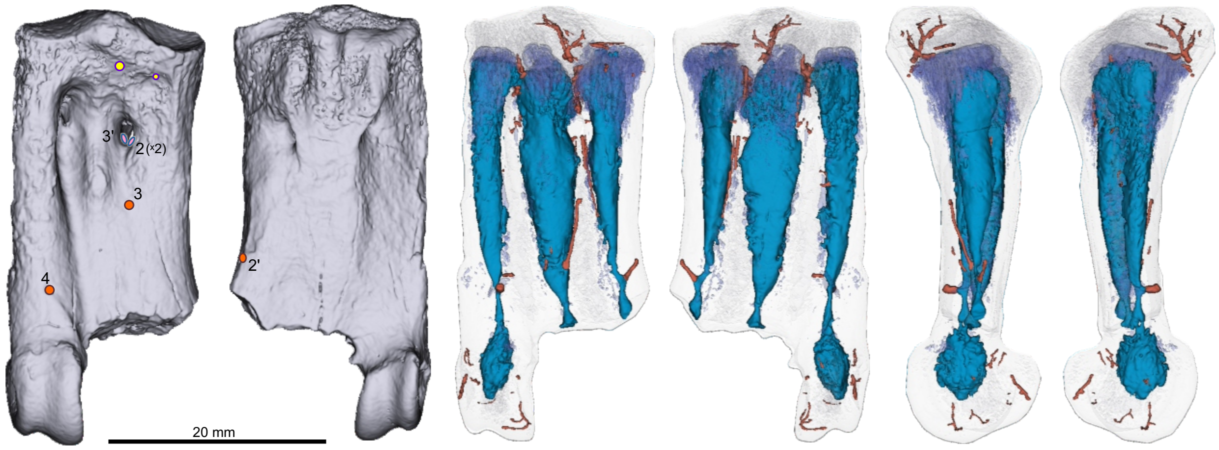

A542 (*Marambiornopsis sobrali*, holotype)

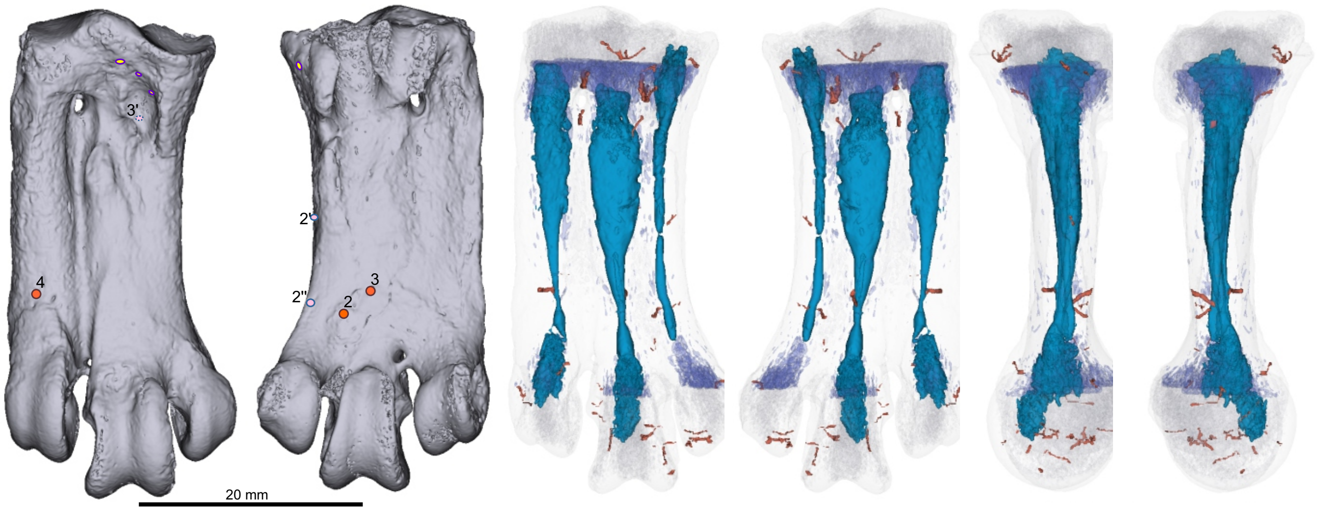

Fig. S1g

AV896268 (*Pygoscelis adeliae*, extant)

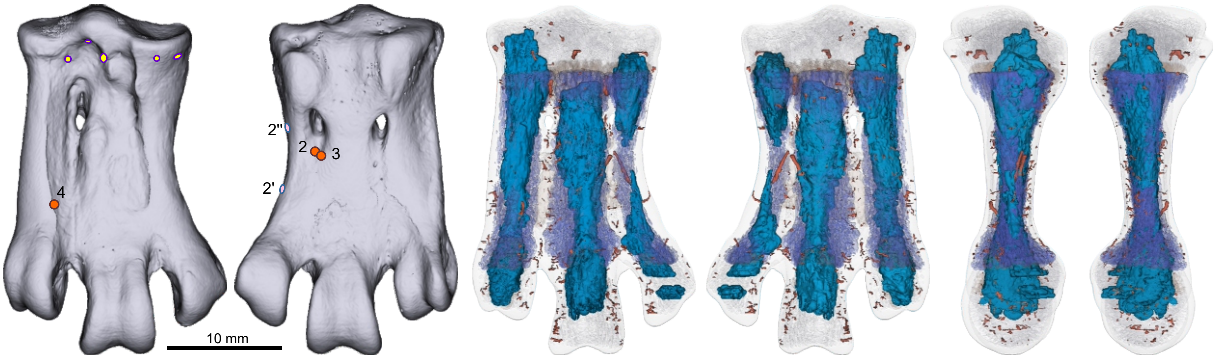

AV928172 (*Aptenodytes patagonicus*, extant; mirrored)

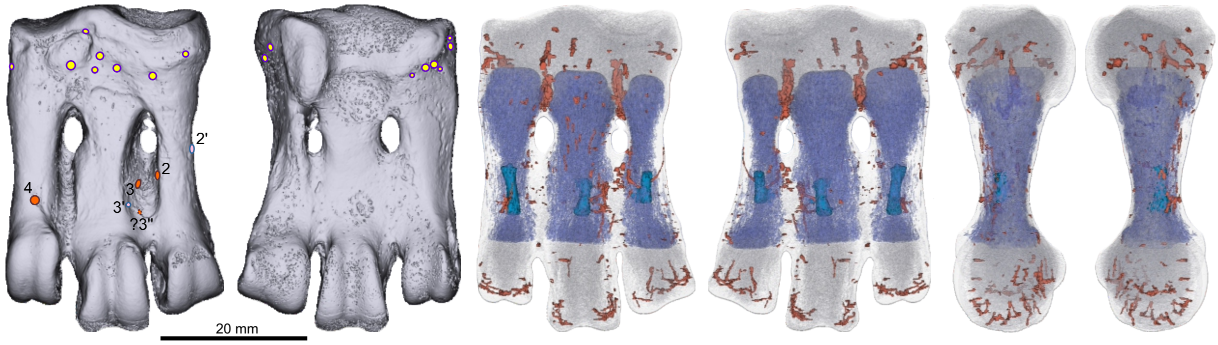

AV20236119 (*Aptenodytes forsteri*, extant, juvenile)

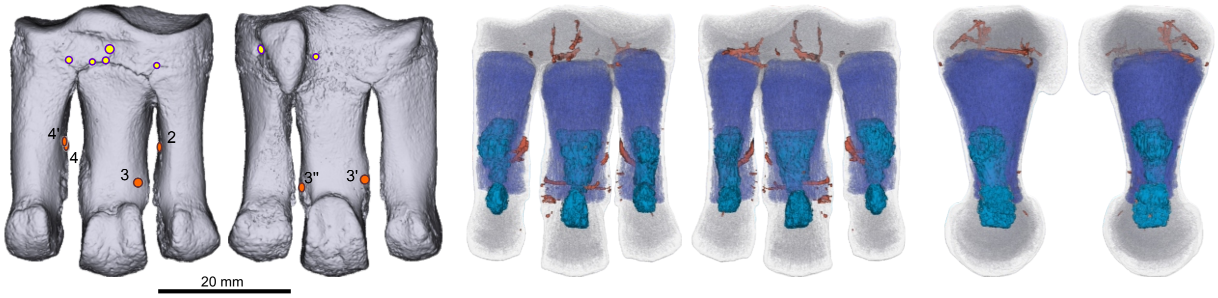

AV677330 (*Aptenodytes forsteri*, extant, adult; mirrored)

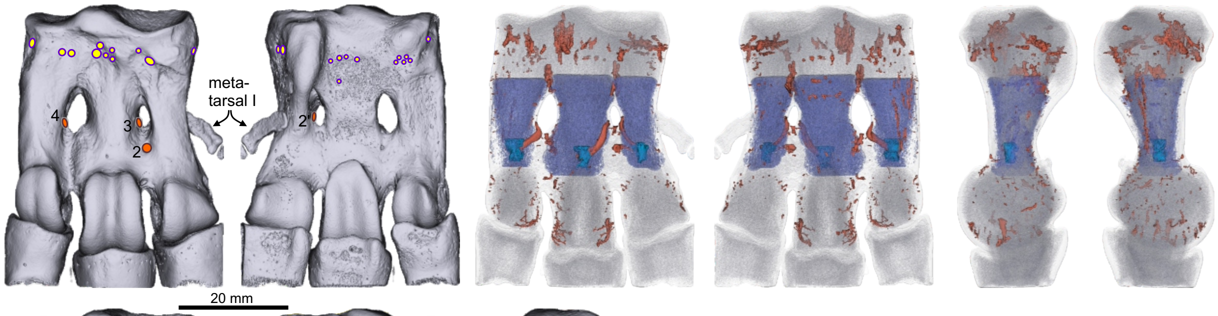

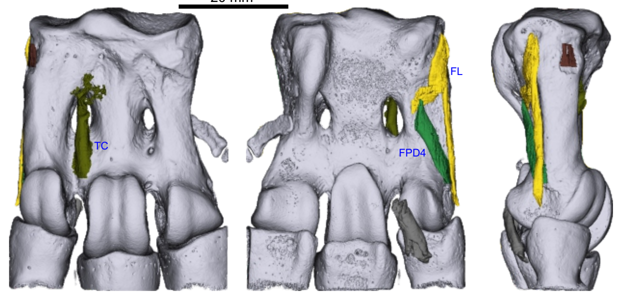

Preserved tendons:

TC *m. tibialis cranialis*
FL *m. fibularis longus* (identification tentative)
FPD4 *m. flexor perforatus digiti IV* (identification tentative)

Fig. S1h

Digital cross-sections of tarsometatarsal shafts

AV896268 (*Pygoscelis adeliae*, extant)

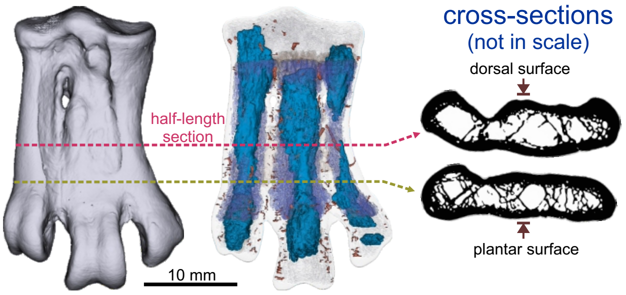

A21 (*Delphinornis larseni*, holotype)

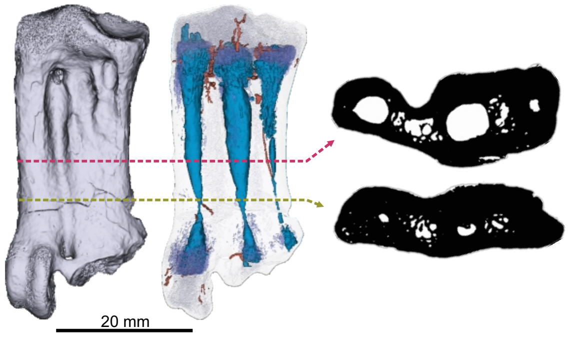

AV928172 (*Aptenodytes patagonicus*, extant)

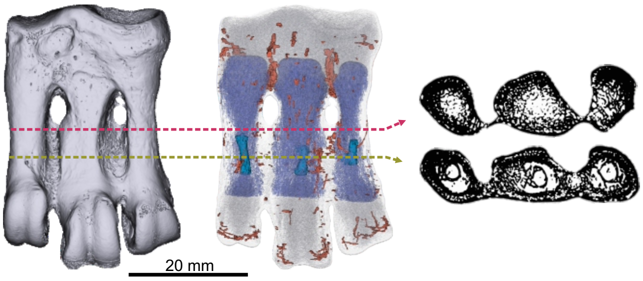

A542 (*Marambiornopsis sobrali*, holotype)

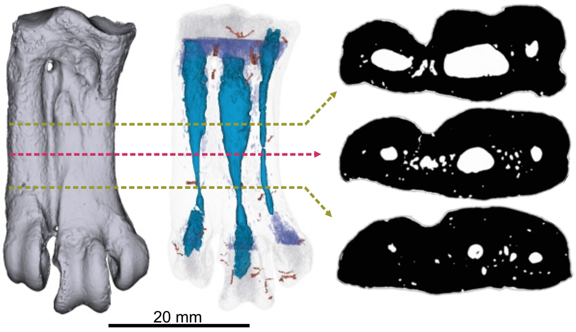

AV677330 (*Aptenodytes forsteri*, extant; adult)

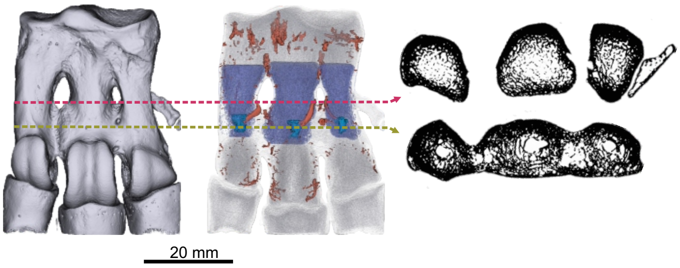

A45 (*Anthropornis nordenskjoeldi*, holotype)

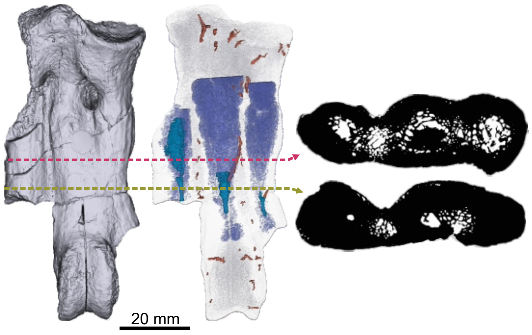

A891 (*Palaeeudyptes* sp.)

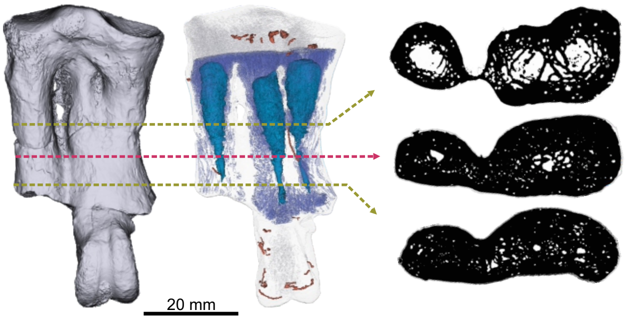

A887 (*Palaeeudyptes* sp.)

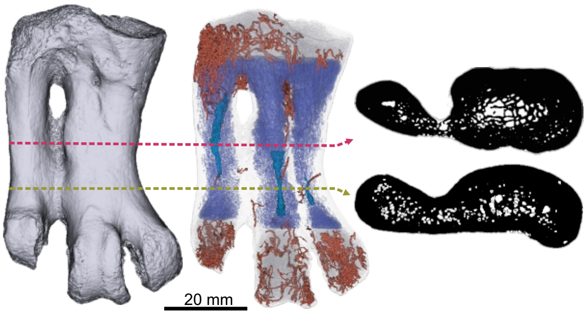

Supplement: Supplementary file 1 — Figure S1 All studied tarsometatarsi of Eocene and extant penguins. [file INZ2-20-551-s001.pdf]
