# Supplementary figures and images for: Fossil and modern penguin tarsometatarsi: cavities, vascularity, and resilience
Source: Integr Zool. 2024 Jun 10;20(3):551–67. doi: 10.1111/1749-4877.12852 (PMC12046465; doi:10.1111/1749-4877.12852)

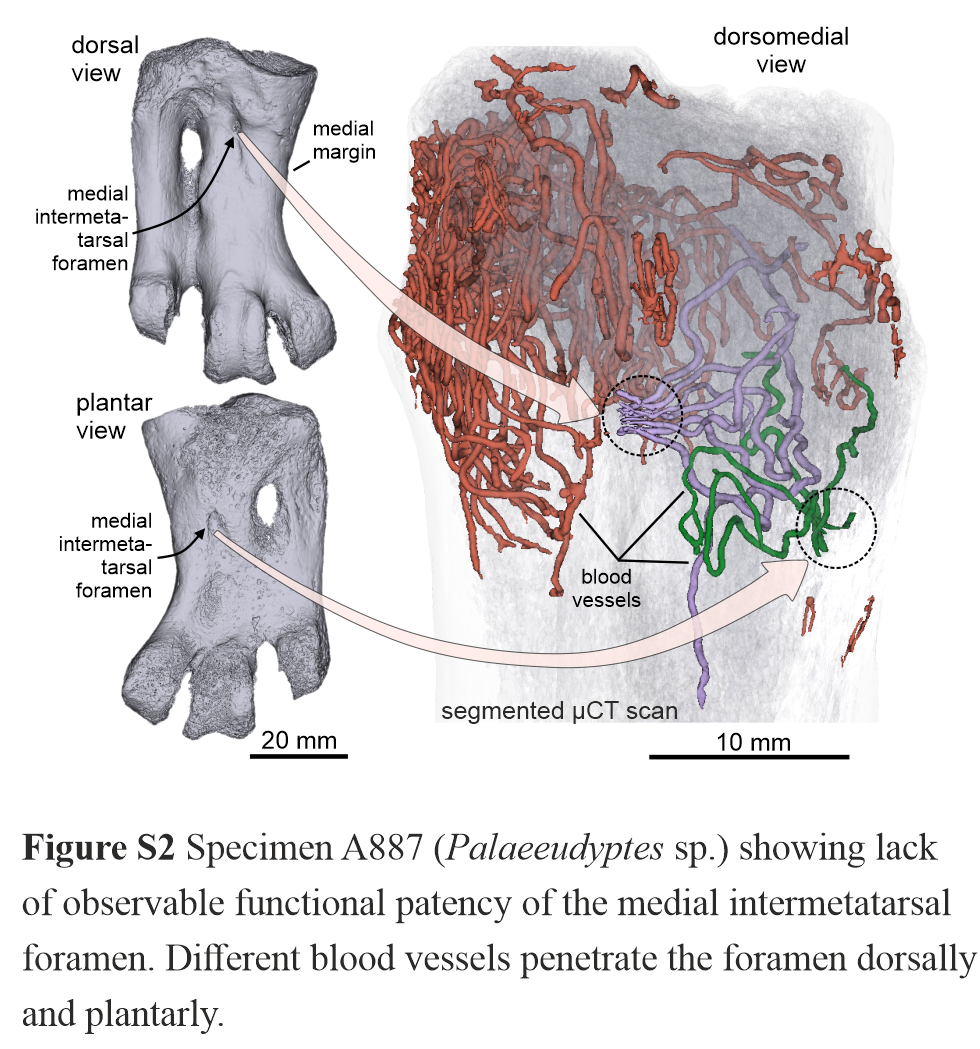

Supplement: Supplementary file 2 — Figure S2 Specimen A887 (Palaeeudyptes sp.) showing lack of observable functional patency of the medial intermetatarsal foramen. Different blood vessels penetrate the foramen dorsally and plantarly. [file INZ2-20-551-s002.tif]

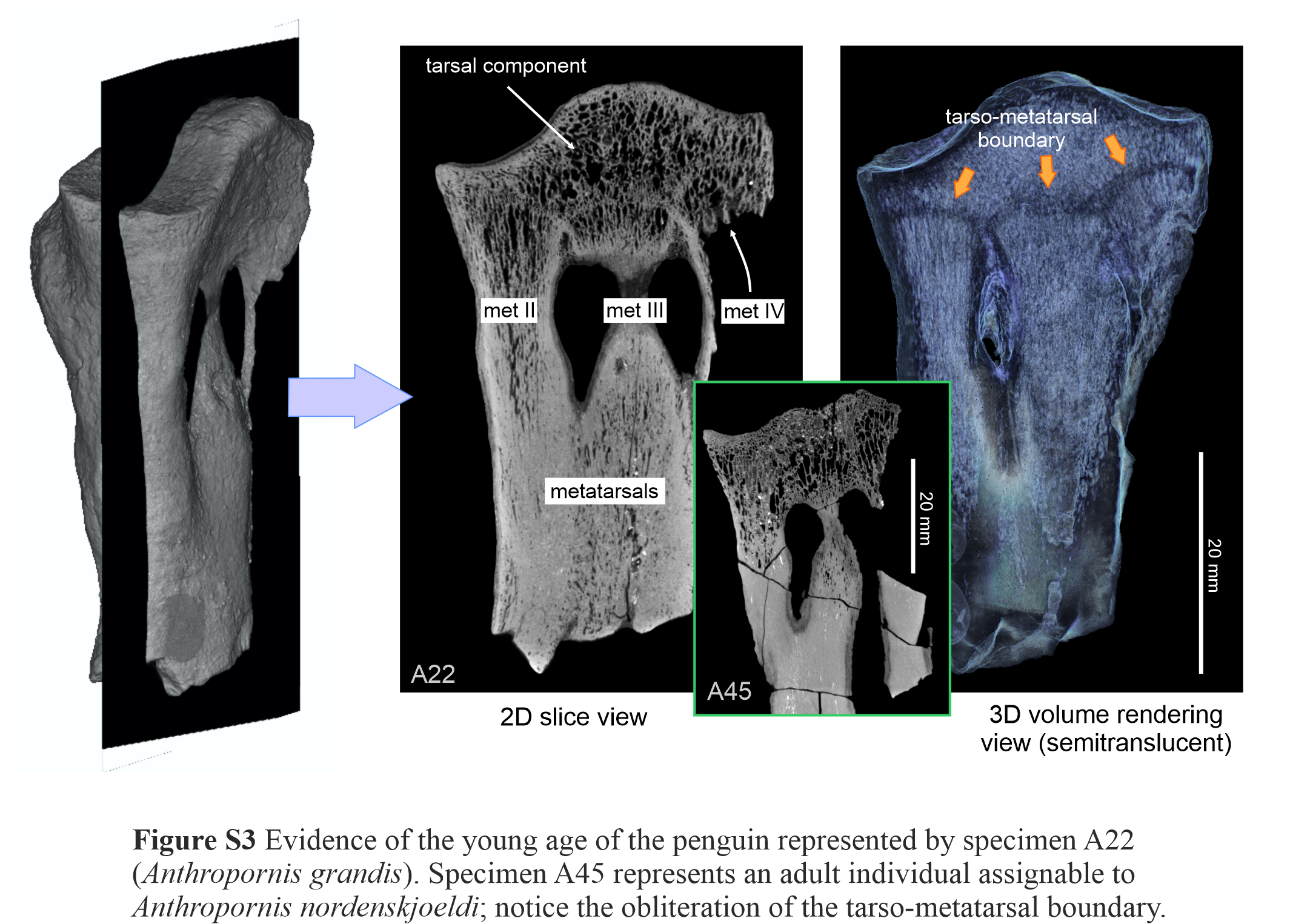

Supplement: Supplementary file 3 — Figure S3 Evidence of the young age of the penguin represented by specimen A22 (Anthropornis grandis, holotype). Specimen A45 represents an adult individual assignable to Anthropornis nordenskjoeldi; notice the obliteration of the tarso‐metatarsal boundary. [file INZ2-20-551-s003.tif]
