# Supplementary material for: Fossil and modern penguin tarsometatarsi: cavities, vascularity, and resilience
Source: Integr Zool. 2024 Jun 10;20(3):551–67. doi: 10.1111/1749-4877.12852 (PMC12046465; doi:10.1111/1749-4877.12852)
Supplement: Supplementary file 4 — Table S1 Measurements of studied penguin tarsometatarsi [file INZ2-20-551-s004.docx]

**SUPPLEMENTARY MATERIALS**

**Table S1** Measurements of studied penguin tarsometatarsi

| Taxon | Specimen ID | Total length  (mm) | Proximal width  (mm) | Midshaft width  (mm) |
| --- | --- | --- | --- | --- |
| ‘Giant’† Eocene penguins |  |  |  |  |
| *Anthropornis grandis* (holotype) | A22 | - | >32 | - |
| *Anthropornis nordeskjoeldi* (holotype) | A45 | 88.8 | - | 37.3 |
| *Anthropornis* sp. | A584 | - | - | 34.8 |
| *Anthropornis* sp. | A901 | - | 36.9 | 30.3 |
| *Palaeeudyptes gunnari (*holotype) | A7 | ~64.4 | - | 26.0 |
| *Palaeeudyptes* sp. | A541 | 71.3 | 36.0 | 32.5 |
| *Palaeeudyptes* sp. | A586 | 73.3 | 37.8 | - |
| *Palaeeudyptes* sp. | A594 | 79.5 | - | 31.3 |
| *Palaeeudyptes* sp. | A815 | 65.5 | 33.2 | 28.2 |
| *Palaeeudyptes* sp. | A821 | 76.7 | - | 29.0 |
| *Palaeeudyptes* sp. | A845 | 72.4 | 34.1 | 28.9 |
| *Palaeeudyptes* sp. | A846 | 69.2 | 36.8 | 30.6 |
| *Palaeeudyptes* sp. | A887 | 75.8 | >36.3 | 31.4 |
| *Palaeeudyptes* sp. | A891 | 68.0 | 35.2 | 27.7 |
| *Palaeeudyptes* sp. | A900 | 76.4 | 41.4 | 34.4 |
| Other Eocene penguins |  |  |  |  |
| *Delphinornis larseni* (holotype) | A21 | - | >19.5 | 16.7 |
| *Delphinornis* sp. | A587 | 47.9 | - | 16.9 |
| *Delphinornis* sp. | A820 | - | 22.3 | 18.9 |
| *Delphinornis* sp. | A861 | 46.5 | 18.7 | 17.2 |
| *Delphinornis* sp. | A869 | - | 17.5 | 16.9 |
| *Marambiornopsis sobrali* | A542 | 43.8 | 17.4 | 15.1 |
| Extant penguins |  |  |  |  |
| *Pygoscelis adeliae* | AV896268 | 31.2 | 15.4 | 13.7 |
| *Aptenodytes patagonicus* | AV928172 | 45.0 | 26.0 | 24.6 |
| *Aptenodytes forsteri* juv. | AV20236119 | 41.2 | 29.6 | 28.9 |
| *Aptenodytes forsteri* | AV677330 | 46.0 | 30.8 | 30.6 |

† This term is commonly applied to fossil penguins whose estimated size exceeds that of the present-day Emperor penguin (*Aptenodytes forsteri*)
